# Involvement of children and young people in the conduct of health research: A rapid umbrella review

**DOI:** 10.1111/hex.14081

**Published:** 2024-06-06

**Authors:** Katherine A. Wyatt, Jessica Bell, Jason Cooper, Leanne Constable, William Siero, Carla Pozo Jeria, Simone Darling, Rachel Smith, Elizabeth K. Hughes

**Affiliations:** ^1^ Murdoch Children's Research Institute Parkville Victoria Australia; ^2^ School of Law University of Warwick Coventry UK; ^3^ Centre for Community Child Health Royal Children's Hospital Parkville Victoria Australia; ^4^ Department of Paediatrics The University of Melbourne Parkville Victoria Australia; ^5^ School of Psychological Sciences The University of Melbourne Parkville Victoria Australia

**Keywords:** adolescent, child, community‐based participatory research, health services research, patient participation, research design

## Abstract

**Background:**

Patient and public involvement and engagement (PPIE) have long been considered important to good research practice. There is growing, yet diverse, evidence in support of PPIE with children and young people (CYP). We must now understand the various approaches to involvement of CYP in research.

**Aims:**

This rapid umbrella review aimed to provide an overview of when, how and to what extent CYP are involved in the conduct of health research, as well as the reported benefits, challenges, and facilitators of involvement.

**Methods:**

We searched OVID Medline, Embase and PubMed. Published reviews were included if they reported meaningful involvement of CYP in the conduct of health research. Extracted data were synthesised using thematic analysis.

**Results:**

The 26 reviews included were predominately systematic and scoping reviews, published within the last decade, and originating from North America and the United Kingdom. CYPs were involved in all stages of research across the literature, most commonly during research design and data collection, and rarely during research funding or data sharing and access. Researchers mostly engaged CYP using focus groups, interviews, advisory panels, questionnaires, and to a lesser extent arts‐based approaches such as photovoice and drawing. Visual and active creative methods were more commonly used with children ≤12 years. The evidence showed a shared understanding of the benefits, challenges, and facilitators for involvement of CYP, such as time and resource commitment and building partnership.

**Conclusion:**

Overall, the review identified consistency in the range of methods and approaches used, and stages of research with which CYP are commonly involved. There is a need for more consistent reporting of PPIE in the literature, both in terminology and detail used. Furthermore, the impact of approaches to CYP involvement on research and community outcomes must be better evaluated.

**Patient/Public Contribution:**

This review forms part of broader research initiatives being led by the authors. Together, these projects aim to support embedding of child voices in research practice and to explore the desirability and suitability of Young Persons Advisory Groups within birth cohort studies. The findings from this review, alongside public and stakeholder consultation, will inform development of resources such as practice recommendations to guide future involvement of CYP in health research undertaken at the author's respective institutions.

## 1 INTRODUCTION

There has been an accelerating shift towards increasing patient and public involvement and engagement (PPIE) in the conduct of research. Although approaches can vary, the key concept is consistent—to foster research carried out ‘with’ or ‘by’ members of the public rather than ‘to’, ‘about’ or ‘for’ them’.^1^ This perspective has been recognised throughout many areas of healthcare, policy and advocacy.^2^

However, evidence for best practice involvement of children and young people (CYP) in these domains has only just begun to take shape. Until recently, their active involvement, and inclusion of their voice, in research has been noticeably less prevalent than with adult cohorts, despite its importance being increasingly recognised.^3^, ^4^, ^5^

The growing, international consensus is that CYP have a right to be involved in decisions that affect them, and that there are distinct implications for how this extends to the conduct of paediatric and youth research.^6^, ^7^, ^8^, ^9^ Involvement in such activities appears to be warranted on both ethical and epistemic grounds. The ethical justification comes from the rights‐based framework of the United Nations Convention on the Rights of the Child which stipulates that children have the right to express their views freely, and that their views ought to be given due weight in all matters affecting them.^10^ Further justification lies with the benefits for researchers, including enhanced relevancy and quality of research findings,^3^ as well as in the enfranchisement of CYP as knowledge agents in their own right and as co‐creators alongside adults. When research is guided by those with lived experience and perspective, outputs become more congruent with the needs and priorities of those impacted.^11^ In the case of CYP, their involvement in research is more likely to produce novel insights that may not otherwise be available through research ‘on’ CYP or where engagement is with adult informants alone.^3^ This in turn supports the development of more effective policies, services and resources to address the needs, issues and barriers identified by, and impacting on, CYP.^12^

A wealth of literature is now emerging on the involvement of CYP in research. It demonstrates the numerous ways that CYP can contribute to research planning, development, implementation and translation.^2^, ^4^ To date, a wide array of methodologies have been used to conduct PPIE with CYP. It is now important to better understand, assess and consider best practice approaches to ensure the adoption of such methods is done meaningfully within research practice.^13^

Although there are prior reviews on the involvement of CYP in health research, there is considerable variability in the scope of literature reviewed and reported by reviews, including the setting, types, and methods of involvement, as well as the purpose and depth of outcomes. This poses a significant challenge for those trying to interpret the literature on this topic. To build on and avoid duplication of existing work, a rapid umbrella review was conducted with the intention of mapping and comprehensively synthesising the wide variety of reviews available in the literature. These findings, in conjunction with public consultation, will inform research initiatives led by the authors with a focus on co‐design and involvement of CYP in research governance. Therefore, the objective of this review was to explore when, how and to what extent CYP are involved in the conduct of health research, as well as the benefits, challenges and facilitators of involvement identified to date.

## 2 MATERIALS AND METHODS

This umbrella review was registered with an International Prospective Register of Systematic Reviews; CRD42023461703. Wherever possible, our methodology and reporting were informed by the Preferred Reporting Items for Systematic Reviews and Meta‐Analyses (PRISMA)^14^ and Cochrane guidelines for overviews of reviews.^15^

We defined the following terms:

- *Children*: All persons aged 12 and under.^16^

- *CYP*: All persons under the age of 25, inclusive of children (<18 years),^10^ adolescents (10–19 years)^17^ and youth (15–24 years).^18^
- *‘Representatives’ of CYP*: Any persons involved alongside CYP who have lived experience or training in caring for CYP. This could include parents/guardians, youth services, teachers, health practitioners, and other relevant experts.
- *Involvement*: research ‘carried out ‘with’ or ‘by’ members of the public (in this case CYP) rather than ‘to’, ‘about’ or ‘for’ them’.^19^ This differs from ‘Participation’, ‘where people take part in a research study’,^19^ except in circumstances where participative activities were designed to give the CYP voice or agency in research (e.g., focus groups for CYP to voice perspectives, photovoice to enable CYP to document their own lived experience).
- *Health research*: Research related to physical and mental health and wellbeing (including observational and interventional research).

### 2.1 Search strategy

The search strategy was developed by the authors with an experienced research librarian. Searches were performed in Ovid MEDLINE, Ovid Embase and PubMed (National Library of Medicine) databases in August 2023. Search terms were grouped based on population (CYP), setting (health research), action (involvement or collaboration), impact (research conduct), and literature type (reviews). The full search is presented in Appendix S1. Additional reviews known by the authors were also included if deemed eligible.

Citations were downloaded into EndNote (Clarivate Analytics) and saved in the Systematic Review Accelerator (SRA).^20^ Duplicate reviews were removed using the SRA Deduplicator (with each duplicate manually verified).

### 2.2 Selection of reviews

Two authors (K. A. W. and J. C.) undertook the title and abstract screening and full‐text review. Review was conducted independently and blinded. The SRA Screenatron tool was used to aid screening.^20^

#### 2.2.1 Inclusion criteria

Searches were limited to reviews only (including systematic reviews, scoping reviews and other literature reviews). No date or language restrictions were applied. Reviews were considered relevant if they reported on (1) involvement of CYP in (2) the conduct of (3) health research. Involvement in personal clinical care and public health initiatives, policies or guidelines were not included unless there was an element of research incorporated (e.g., evaluation and dissemination of results). Reviews that included adults (>24 years) were only eligible if CYP (≤24) was the primary focus.

#### 2.2.2 Exclusion criteria

Reviews/studies were excluded if (1) CYP (and/or their representatives) were involved only as research participants, (2) there was sufficient detail to indicate that involvement was ‘tokenistic’ (Tokenism, in this context, is defined as the practice of making perfunctory or symbolic efforts to involve CYP in the conduct of health research [modified from Hahn et al.^21^]. For example, an attempt at PPIE with CYP could be tokenistic if their input is not fairly considered, unnecessarily limited or highly controlled by adults, whether intentional or not) and (3) insufficient description of the methods and/or nature of involvement was provided (e.g., necessary details could not be extracted). Due to the rapid nature of this umbrella review, reviews were also excluded if the full text was not accessible through the Royal Children's Hospital or the University of Melbourne libraries.

### 2.3 Data extraction and synthesis

Data extraction included reference (author, year), review type, setting and objectives, data available at the individual source study level (region, quantity and age of those involved, the stage, method, and level of involvement), as well as data available at the review level (benefits, challenges and facilitators of involvement).

Two authors (K. A. W. and J. C.) conducted extraction and synthesis independently, with data checked for accuracy by the alternate reviewer. A combined approach of content (individual source study variables) and thematic (review level variables) analysis was used to overcome heterogeneity in reporting of results. Data were coded and categorised to enable comparison and analysed in whole (i.e., ‘CYP and/or their representatives’) and age‐defined subgroups, ‘CYP alone’ (i.e., ≤24 years of age only, no representatives) and ‘children alone’ (i.e., ≤12 years of age only, no representatives).

**Figure 1:**
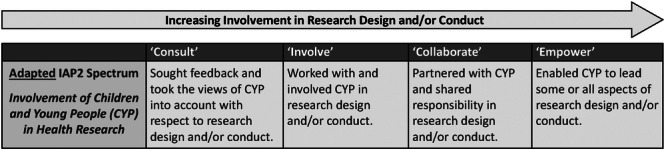
Level of involvement of children and young people in health research. Adapted from the International Association of Public Participation (IAP2) Public Participation Spectrum^23^ with influence from Shier's ‘Pathways to participation’ model.^24^

An adapted taxonomy proposed by Haijes and van Thiel^22^ was used to capture method types for involvement. This involves categorisation of participatory methods into four groups (verbal, written, visual and active), as well as ‘combination’ which further captures where multiple of these methods were used in conjunction. Similarly, a modified version of the International Association of Public Participation (IAP2) Public Participation Spectrum was used to infer the ‘level of involvement’ reported for studies within reviews (Figure 1).^23^ The IAP2 captures the depth and meaningfulness of involvement across a spectrum. Data were only included if sufficient detail was reported and/or an existing model was used by reviews to assess level of involvement. The ‘observation’ category of Haijes and van Thiel^22^ and the ‘inform’ level of IAP2 were not considered meaningful involvement in the context of this review and were therefore not included in the results.

Data were extracted from reviews for original research studies only. Further information was sought from source studies if required and available. Primary publication overlap was assessed using a citation matrix,^15^ and the ‘corrected covered area’ (CCA), a measure of the overlap within the reviews of an umbrella review, was calculated as 0.49%. Pieper et al.^25^ consider a CCA value lower than 5 as a slight overlap, therefore we suggest that a CCA < 0.5% indicates that limited biasing of results is likely to have occurred as a result of the minimal overlap. Therefore, duplicate primary publications across reviews were not removed; however, data were consolidated in instances where multiple source articles were referenced for the same study within a review.

### 2.4 Quality assessment

Two authors (K. A. W. and J. C.) undertook Risk of Bias assessment for included systematic reviews (*n* = 12) using the ROBIS Tool as this was determined most appropriate for this umbrella review.^26^ There was no known established tool for appraising the quality of scoping reviews. However, the PRISMA extension for scoping reviews (PRISMA‐ScR) checklist was used to determine whether included scoping reviews (*n* = 10) adhered to essential reporting items.^27^ Remaining reviews (*n* = 4) were not appraised due to the absence of an appropriate tool.

Finally, disagreement during screening and selection, data extraction, and risk of bias assessment were resolved by consensus between two authors (K. A. W. and J. C.).

## 3 RESULTS

### 3.1 Search and screening results

**Figure 2:**
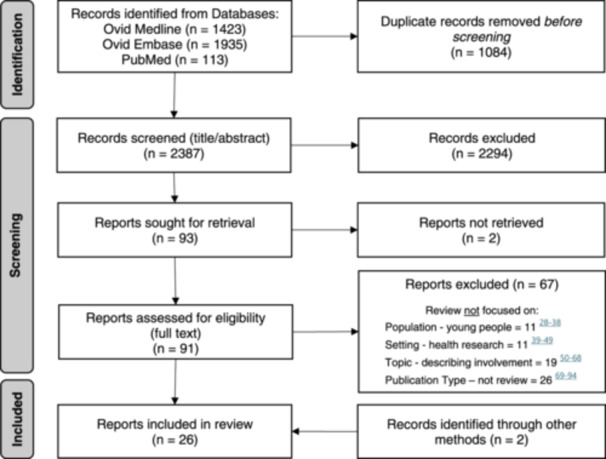
Search and screening process. Adapted from the Preferred Reporting Items for Systematic Reviews and Meta‐Analysis 2020 flow diagram template.^14^, ^28^, ^29^, ^30^, ^31^, ^32^, ^33^, ^34^, ^35^, ^36^, ^37^, ^38^, ^39^, ^40^, ^41^, ^42^, ^43^, ^44^, ^45^, ^46^, ^47^, ^48^, ^49^, ^50^, ^51^, ^52^, ^53^, ^54^, ^55^, ^56^, ^57^, ^58^, ^59^, ^60^, ^61^, ^62^, ^63^, ^64^, ^65^, ^66^, ^67^, ^68^, ^69^, ^70^, ^71^, ^72^, ^73^, ^74^, ^75^, ^76^, ^77^, ^78^, ^79^, ^80^, ^81^, ^82^, ^83^, ^84^, ^85^, ^86^, ^87^, ^88^, ^89^, ^90^, ^91^, ^92^, ^93^, ^94^

The search yielded 3471 articles, of which 2387 were screened after removing duplicates. A further 2294 were removed during title/abstract screening. Of the 91 full‐text reviews available, 24 met the inclusion criteria and an additional 2 eligible reviews were identified by hand‐searching during preliminary scoping of the literature. In total, 26 reviews were included in this rapid umbrella review. The full search and screening process is presented in Figure 2.

### 3.2 Characteristics of included reviews

Characteristics of the reviews are collated in Table 1. Review type was largely systematic (*n* = 12; 46%) and scoping (*n* = 10; 38%) reviews, all of which were published within the last decade (2013–2023). Reviews were primarily focused on general health research (*n* = 14; 54%), as well as specific conditions (*n* = 4; 16%), such as mental health (*n* = 2; 8%), or populations (*n* = 3; 12%).

**Table 1:** Characteristics of included reviews.

Reference	Review type	Setting	No. of studies^a^	Review period	Objectives
Ali et al.^95^	Scoping review	Mental health	16	2005–2021	Identify approaches to child, adolescent and youth engagement in mental health studies as well as study‐reported barriers, constraints and facilitators to engagement.
Bailey et al.^96^	Systematic review	Specific population	8	1990–2015	Investigate how children with a disability have been involved as research partners; specifically, how they have been recruited, the practicalities and challenges of involvement and how these have been overcome and impacts of involvement for research, and disabled CYP.
Bakhtiar et al.^3^	Systematic review	General health	25	2010–2021	Investigate the paradigms and the discourses around research with or by children; what others are doing in the field and the challenges and the benefits of researching in this way.
Branquinho, et al.^97^	Systematic review	General health	12	Up to 2020	Investigate the characteristics of community‐based YPAR programmes with a focus on health and well‐being and their recommendations for future programmes.
Dubois et al.^98^	Scoping review	Social research	17	NR	Identify specific ethical issues and ways of overcoming challenges in conducting research with children on some aspects of their family lives.
Flynn et al.^99^	Scoping review	General health	17	1946–2019	Comprehensively map current patient engagement strategies with parents and families across existing published paediatric health research literature.
Fountain, et al.^100^	Systematic review	General health	43	2010–2019	Investigate photovoice projects conducted with youth in the United States over the past 10 years (2010–2019).
Freire et al.^5^	Systematic review	General health	26	2000–2020	Synthesise the methods and approaches used to enable children, adolescents and families to be involved in a participatory approach in research conducted to inform development of health resources and interventions aimed at children and adolescents.
Gibbs et al.^101^	Systematic review	Technology in health research	5	2000–2018	Provide insights into the use of technology to scale YPAR.
Haijes et al.^22^	Systematic review	General health	9	Up to 2014	Describe and assess the available knowledge of participatory methods in paediatric research.
Hunleth et al.^102^	Scoping review	Disease prevention	114	2007–2018	Examine the type, extent and meaningfulness of children's participation in qualitative health intervention research.
Larsson et al.^103^	Scoping review	General health	41	Up to 2017	Systematically map recent research involving CYP in the development of interventions targeting issues of health and well‐being.
Mandoh et al.^104^	Systematic scoping review	Disease prevention	71	1992–2020	Analyse the current modes and nature of adolescent participation in obesity prevention research decision‐making.
McCabe et al.^9^	Systematic review	General health	16	2000–2023	Describe the impacts of youth engagement on mental health research and to summarise youth engagement in mental health research.
McNeill et al.^105^	Systematic review	Patient‐ reported outcomes	35	2009–2018	Assess child and family engagement in the selection of patient‐reported outcomes for clinical studies/clinical settings and development of PROMs/PREMs across the paediatric literature.
Nathan et al.^106^	Systematic review	Specific population	24	2009–2021	Synthesise and examine the experience and use of arts‐based methodologies with young people with complex psychosocial needs.
Nortvedt et al.^107^	Thematic synthesis	General health	7	2010–2020	Synthesise existing literature on how young people's involvement in coproduction can contribute to better welfare services.
Racine et al.^13^	Narrative review	Specific population	5	NR	Identify gaps in youth engagement methods, barriers to youth engagement, and approaches for increasing youth engagement methods within the field of child maltreatment research.
Rouncefield‐Swales et al.^108^	Scoping review	General health	40	2000–2019	Identify, synthesise and present what is known from the literature about PPI and engagement activities with CYP in health‐related research.
Sellars et al.^109^	Scoping review	General health	15	2019 only	Provide systematic evidence on the methods and impacts of YPAGs in youth‐focused health research.
Sposito et al.^110^	Integrative review	Specific condition	15	2000–2010	Identify playful resources used in qualitative research data collection with child cancer patients, and their forms of application.
Thomas et al.^111^	Narrative review	General health	15	Up to 2021	Investigate and synthesise existing literature on the involvement of CYP as partners in health research.
Valdez et al.^112^	Systematic review	Specific condition	15	1988–2018	Summarise published evidence regarding YPAR for youth substance use prevention; the level of youth engagement and methodologies used in the research process.
van Schelven et al.^113^	Scoping review	General health	22	1990–2019	Gain insight into developments in the existing literature on PPI of young people with a chronic condition by mapping reported definitions, goals, activities, experiences and impact.
Vanderhout et al.^114^	Scoping review	General health	25	Up to 2021	Identify impacts of patient and family engagement in child health research on the research process, research teams and patient and family partners.
Vaughn et al.^115^	Scoping review	General health	33	1985–2012	Review published studies that use an authentic CBPR approach in child health to highlight the benefits, barriers, and scope of this approach with paediatric populations.

Abbreviations: CBPR, community‐based participatory research; CYP, children and young people; NR, not reported; PPI, patient and public involvement; PROMs/PREMs, patient‐reported outcome/experience measures; YPAG, young persons advisory group; YPAR, youth‐led participatory action research.

a Relevant source articles only, excluding publications within reviews that report on the same research study/project data.

The 26 reviews reported on 671 uniquely relevant studies of varying designs, with an average of 26 studies per review (range of 5–114). Together, the reviews screened for articles published from all time to 2023. However, most were published within the last 10 (*n* = 427; 64%) to 20 years (228; 34%) (Table 2).

**Table 2:** Summary of source studies.

Characteristic (*n* = studies with available data)	Subcategory	*N* (%) of studies
Publication year (*n* = 670)	2013–2023	427 (64%)
	2003–2012	228 (34%)
	1993–2002	14 (2%)
	<1993	1 (0.2%)
Region (*n* = 671)	North America	322 (48%)
	United Kingdom	159 (24%)
	Europe	80 (12%)
	Australia and New Zealand	51 (8%)
	Africa	17 (3%)
	South America	18 (3%)
	Asia	15 (2%)
	NR or other	9 (1%)
Stakeholder^a^ (*n* = 671)	CYP	635 (95%)
	Representatives^b^	223 (33%)
Number of CYP (*n* = 369)	1–5	36 (10%)
	6–10	94 (25%)
	11–20	86 (23%)
	21–50	93 (25%)
	51–100	32 (9%)
	100+	28 (8%)
	Median (range)	17 (1–2100)
Age groups of CYP (years)^a^ (*n* = 462)	Studies with *at least one* CYP in age group^a^	
	Children (≤12)	297 (64%)
	Children (<18)	437 (95%)
	Adolescent (10–19)	441 (95%)
	Youth (15–24)	298 (65%)
	Young adult (>24)^b^	42 (9%)
	Studies with *all* CYP within age range^a^	
	Children (≤12)	99 (21%)
	Children (<18)	261 (56%)
	CYP (≤24)	420 (91%)
	Young adult (>24)	0 (0%)
Minimum age (*n* = 477)	Mean (SD; range)	11 (4.0; 2–21)
Maximum age (*n* = 463)	Mean (SD; range)	17 (4.8; 3–30)
Average age (*n* = 478)	Mean (SD; range)	14 (4.1; 2–30)
Number of representatives (*n* = 86)	1–5	16 (19%)
	6–10	13 (15%)
	11–20	26 (30%)
	21–50	22 (26%)
	51–100	7 (8%)
	100+	2 (2%)
	Median (range)	16 (2–118)
Types of representatives^a^ (*n* = 206)	Parents/guardians	124 (60%)
	Health/clinical experts	33 (16%)
	Youth services	14 (7%)
	Research experts	5 (2%)
	Other (e.g., teachers)	30 (15%)

Abbreviations: CYP, children and young people; NR, not reported.

^a^ Multiple selections possible.

^b^ All studies in this category also included CYP (≤24 years).

### 3.3 Characteristics of source studies

Source studies predominantly originated from North America (*n* = 322; 48%), followed by the United Kingdom (*n* = 159; 24%), Europe (*n* = 80; 12%) and Australia and New Zealand (*n* = 51; 8%). Involvement was described for CYP in 635 (95%) studies, and for representatives in 223 (33%), with 192 (29%) of studies describing the involvement of both (Table 2).

The number of CYP involved was only available for 369 (58%) studies, with a median of 17 CYP per study. The age range for CYP involved was available for 462 (73%) studies, covering children ≤12 (*n* = 297; 64%), children < 18 (*n* = 437; 95%), adolescents (10–19 years; *n* = 441; 95%) and youth (15–24 years; *n* = 298; 65%). The overall mean age was 14 years (SD: 4.1; range 2–30).

Parents/guardians/caregivers were the most common representative type involved in studies alongside CYP (*n* = 124; 60%). The number of representatives was only available for 86 (38%) studies, with a median of 16 per study.

### 3.4 Stages, methods and level of involvement in research

#### 3.4.1 Research stages

**Figure 3:**
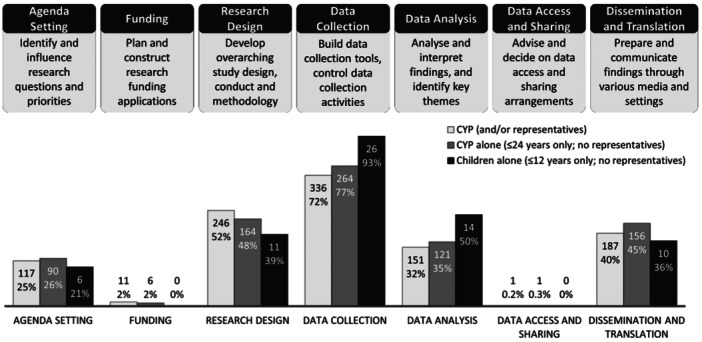
Stage of research involvement (*n*, % of studies with available data). Light grey bars represent combined results for involvement of CYP and/or their representatives (*n* = 469 studies). Dark grey bars represent results for CYP alone (≤24 years old; no representatives; *n* = 344 studies). Black bars represent results for children alone (≤12 years old, no representatives; *n* = 28 studies). NB: multiple selections possible. CYP, children and young people.

Stage of research involvement was available for 469 (70%) studies, with most (*n* = 344; 73%) describing the stage of involvement for CYP alone (i.e., ≤24 years of age only; no involvement of representatives). The definition of involvement at each stage is presented in Figure 3.

Studies most frequently described involvement of CYP (and/or their representatives) in data collection (*n* = 336; 72%) and research design (*n* = 246; 52%). For the purpose of this review, ‘data collection’ refers to instances whereby CYP were involved in building data collection tools (e.g., developing a survey), conducting data collection (e.g., interviewing their peers) or were supported to have meaningful control over the data collection process (e.g., participative activities that enabled CYP voice and expression). Only 11 (2%) studies described involvement in research funding and 1 (0.2%) in data access and sharing.

Over half (*n* = 280; 60%) the studies reported involvement in multiple research stages.

The proportion of involvement was slightly elevated for CYP alone in agenda setting (*n* = 90; 26%), data collection (*n* = 264; 77%), data analysis (*n* = 121; 35%) and dissemination and translation (*n* = 156; 45%) compared to the combined results.

A smaller number of studies reported on the involvement of children aged 12 and under only (*n* = 28; 6%). Children (≤12 years old, no representatives) were more commonly involved in data collection (*n* = 26; 93%) and analysis (*n* = 14; 50%), less in research design (*n* = 11; 39%) and dissemination and translation (*n* = 10; 36%) and not at all in funding or data access and sharing (*n* = 0; 0%).

#### 3.4.2 Research methods

**Figure 4:**
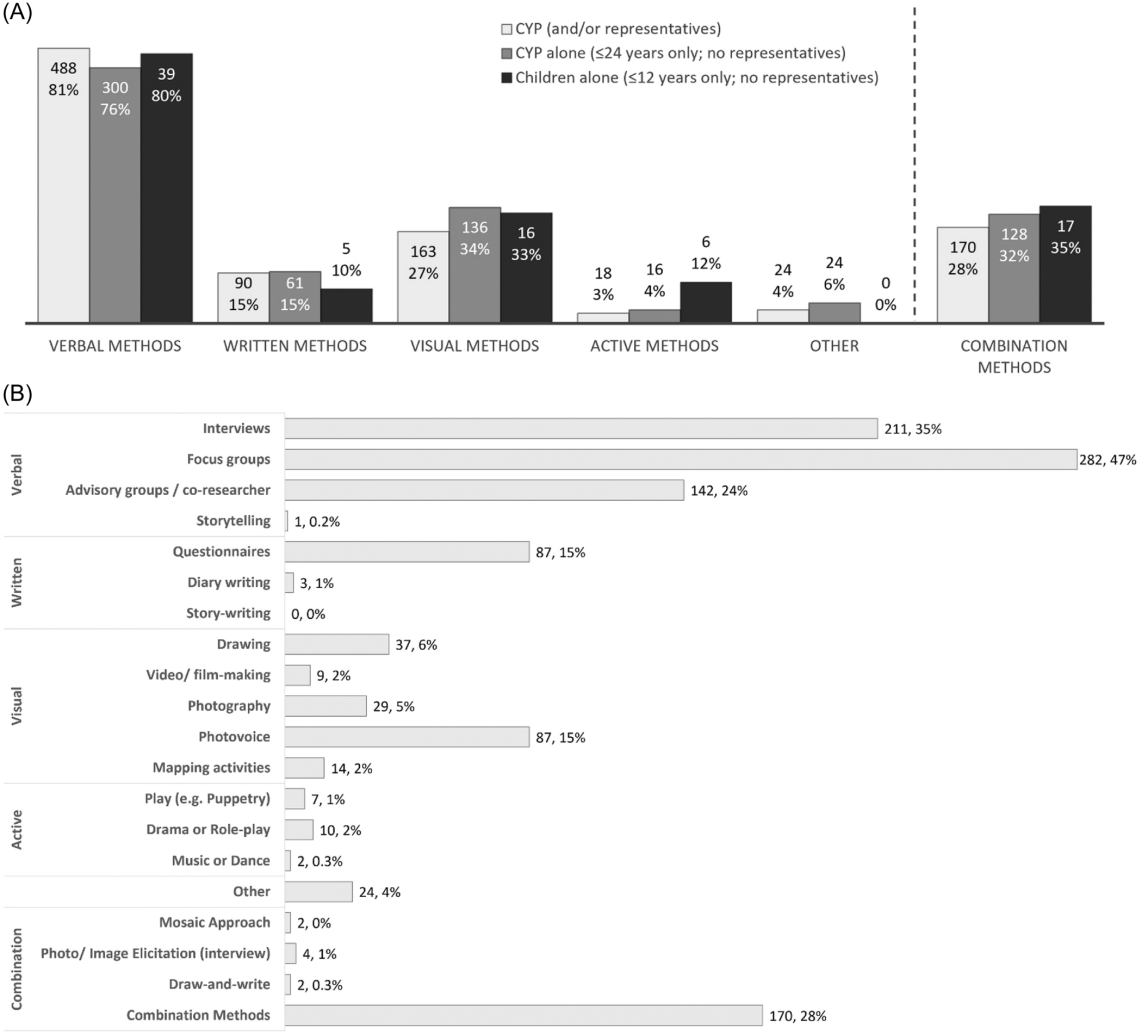
(A) Overarching method types and (B) specific methodologies used for research involvement (*n*, % of studies with available data). Light grey bars represent combined results for involvement of CYP and/or their representatives (*n* = 600 studies). Dark grey bars represent results for CYP alone (≤24 years old; no representatives; *n* = 397 studies). Black bars represent results for children alone (≤12 years old, no representatives; *n* = 49 studies). NB: multiple selections possible. CYP, children and young people.

Methods of involvement were available for 600 (89%) of the studies (Figure 4), with two thirds (*n* = 397; 66%) describing the method used for CYP alone (i.e., ≤24 years of age only; no representatives). Complete data for the method of involvement is tabulated in Appendix S2.

CYP (and/or their representatives) were most often involved through verbal methods (*n* = 488; 81%), such as focus groups (*n* = 282; 47%), interviews (*n* = 211; 35%) and/or advisory groups/co‐researcher roles (*n* = 142; 24%). This was followed by visual methods (*n* = 163; 27%), such as photovoice (*n* = 87; 15%) and drawing (*n* = 37; 6%). Written methods (*n* = 90; 15%) and active methods (*n* = 18; 3%), such as play or drama, were the least frequently used. Almost half (*n* = 258; 43%) of the studies reported using more than one involvement activity, and nearly a third (*n* = 170; 28%) used a combination of method types (e.g. verbal and visual activities).

Methods of involvement for CYP alone were similar, largely facilitated through verbal methods (*n* = 300; 76%), such as focus groups (*n* = 162; 41%) and interviews (*n* = 118; 30%); visual methods (*n* = 136; 34%), such as photovoice (*n* = 80; 20%), and drawing (*n* = 25; 6%) and written methods (*n* = 61; 15%), such as questionnaires/surveys (*n* = 59; 15%). Active methods were proportionately more commonly utilised with children ≤ 12 years old alone (*n* = 6; 12%). ‘Other’ methods (*n* = 24; 6%) largely captured instances of peer mentorship and advocacy in research implementation (e.g., CYP working with researchers to guide and support their peers taking part in the research).

#### 3.4.3 Level of involvement

**Figure 5:**
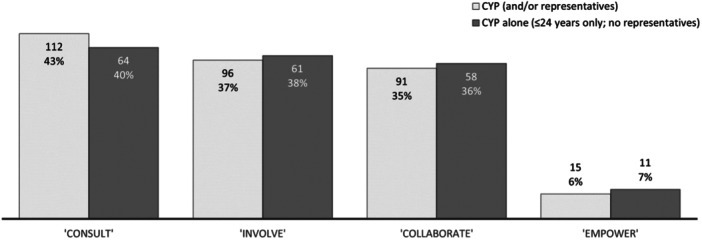
Level of research involvement (*n*, % of studies with available data). Light grey bars represent combined results for involvement of CYP and/or their representatives (*n* = 259 studies). Dark grey bars represent results for CYP alone (≤24 years old; no representatives; *n* = 160 studies). NB: multiple selections possible. CYP, children and young people.

A modified version of the IAP2 spectrum was used to infer ‘level of involvement’ (Figure 1). This was only available for 259 (39%) of the studies (Figure 5), with two thirds (*n* = 160; 62%) describing the level of involvement employed for CYP alone (i.e., ≤24 years of age only; no representatives).

Researchers mostly consulted (*n* = 112; 43%), involved (*n* = 96; 37%) and collaborated (*n* = 91; 35%) with CYP (and/or their representatives). A small proportion (*n* = 43; 17%) of studies reported more than one level of involvement due to varying involvement approaches used. CYP (and/or their representatives) were rarely empowered to conduct research (*n* = 15; 6%), and neither were CYP alone (*n* = 11; 7%).

### 3.5 Benefits of involvement in research

**Figure 6:**
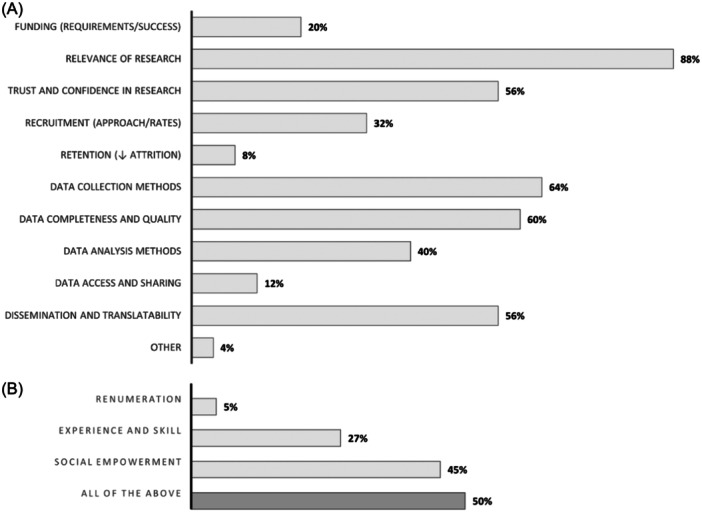
Benefits of involvement reported to positively impact (A) the research study (*n* = 25 reviews) and (B) the CYP involved in the research (*n* = 22 reviews). % of reviews with available data. NB: multiple selections possible. CYP, children and young people.

A range of benefits were identified relating to involvement of CYP in research. Benefits of involvement were reported to positively impact the research study (25 reviews [96%]; Figure 6A) as well as the CYP involved in the research (22 reviews [85%]; Figure 6B).

Involvement of CYP (and/or their representatives) was described to have improved, most prominently, the relevance of the research (*n* = 22, 88%), data collection methods (*n* = 16, 64%), data completeness and quality (*n* = 15, 60%), dissemination and translatability of research findings (*n* = 14, 56%) and trust and confidence of CYP and the community in research (*n* = 14, 56%). Furthermore, CYP involved were reported to have benefited from social empowerment (*n* = 21; 95%), experience and skill development (*n* = 17; 77%) and financial renumeration or gifts (n = 12; 55%).

### 3.6 Barriers and facilitators of involvement in research

Challenges and barriers to involvement of CYP in research were discussed in 25 (96%) reviews, often accompanied by facilitators to improve involvement. Several themes emerged including barriers and facilitators relating to meaningful involvement (*n* = 20; 80%), research commitment (*n* = 21; 84%), trust and partnership (*n* = 21; 84%), accessibility (*n* = 16; 64%), maintenance of engagement (*n* = 16; 64%), recruitment (*n* = 12; 48%), representation (*n* = 10; 40%) and conflict navigation (*n* = 12; 48%). These themes are outlined in Table 3.

**Table 3:** Barriers, challenges and associated facilitators for involvement of CYP.

Barriers and challenges (*N*; %)	Facilitators (summarised from reviews and extended literature)
Research commitment (21; 84%) Limited resources and experience Time constraints Institutional and ethical considerations and restrictions	Plan for youth involvement as early as possible in the research development process.^35^, ^76^, ^108^ Secure funding with realistic expectations of resource and time requirements.^96^ Prepare for institutional and ethical considerations and restrictions, particularly where youth involvement requires specific consent and support needs.^13^, ^72^, ^76^, ^96^, ^101^, ^107^, ^108^, ^113^, ^116^, ^117^ Researchers should have experience or seek training in best‐practice engagement.^13^, ^72^, ^76^, ^96^, ^101^, ^107^, ^108^, ^113^, ^116^, ^117^ Alternatively, experts can be outsourced to lead and guide youth involvement in research.^9^
Trust and partnership (21; 84%) Power differentials and distrust Vulnerability and exploitation	Minimise power imbalances between youth and researchers. Be receptive and respectful to all input provided by CYP.^97^, ^113^ Build genuine, personal relationships and rapport with youth.^75^, ^77^, ^95^, ^99^ Foster symbiotic partnerships through equality, trust and gratitude.^9^, ^102^, ^110^ Establish supportive environments (e.g., settings that are familiar like community or school locations) and practice honest and clear, two‐way communication.^13^, ^56^, ^95^
Meaningful Involvement (20; 80%) Tokenism and ineffective collaboration Misinformation and bias (infantilisation and assumed incompetence)	Involve CYP in all stages of research and facilitate true co‐production and partnership wherever possible.^97^, ^115^ Empower CYP to share responsibility and decision‐making in research.^9^ Offer training and mentorship to CYP to enhance their opportunities and abilities to contribute meaningfully to research.^5^, ^13^, ^114^ Allow dedicated time for reflection and evaluation to learn and adapt future experiences and approaches.^5^, ^13^, ^114^
Accessibility for CYP (16; 64%) Financial constraints Scheduling and availability Location and transportation	Flexibility is required to accommodate the needs of CYP. Use of hybrid approaches (face‐to‐face, online, social media) and suitable timing, frequency and location.^9^, ^13^, ^35^, ^76^, ^77^, ^87^, ^95^, ^96^, ^99^, ^101^, ^115^ Understand that CYP have their own lives and accept fluctuation in ability and willingness to be involved.^97^, ^107^, ^108^, ^115^ Ensure compensation is adequate to not only reimburse expenses (travel, parking, food, accommodation) but also renumerate for time and skills.^13^, ^75^, ^77^, ^96^, ^104^, ^113^, ^115^, ^117^
Maintenance of Engagement (16; 64%) Loss of interest Dwindling motivation Knowledge barriers Mental and physical fatigue	Allow reasonable time and effort to design engaging tasks and activities for CYP.^22^ Ensure approaches are age appropriate, language accessible and tailored to the interests of those involved.^5^, ^73^, ^104^, ^110^ Encourage CYP to guide their own experiences.^5^, ^9^, ^96^, ^107^, ^109^, ^113^ Create safe spaces, frequent breaks and incorporate unrelated socialisation activities.^104^, ^115^ Give back to CYP by assisting with skill attainment and offering new experiences and opportunities.^75^, ^86^, ^99^, ^107^, ^113^ Provide regular feedback on the impact of their contributions and fair recognition of their input to the research.^75^, ^86^, ^99^, ^107^, ^113^
Recruitment (12; 48%) + representation (10; 40%) Small sample size and attrition Lack of diversity and Group polarisation	Ensure recruitment efforts yield a sufficient number of CYP to maintain sample size and account for attrition over time.^13^, ^100^, ^115^ Expanded eligibility and/or targeted recruitment strategies can be useful,^95^ as well as leveraging pre‐established relationships and partner organisations.^100^
Conflict navigation (12; 48%) Shared understanding Differences in opinion Feasibility limits Gatekeepers	Set and manage expectations with CYP from the beginning.^5^, ^22^, ^35^, ^72^, ^76^, ^87^, ^115^, ^118^ Formal/informal agreements or memorandums of understanding can help to establish shared purpose and responsibilities.^112^ Consider nominating a third‐party liaison to mediate discussions.^13^ Demonstrate how all input has been considered and be transparent with any feasibility barriers along the way.^9^, ^107^ Gatekeepers of CYP (e.g., parents/guardians, teachers, carers, etc.) must be kept informed and/or involved with their own separate role.^9^, ^72^, ^87^, ^107^ It is important to establish clear boundaries to prevent adults from dominating involvement.^9^, ^107^

Abbreviation: CYP, children and young people.

### 3.7 Risk of bias/quality assessment

Overall, half of the systematic reviews were assessed as low risk of bias (*n* = 6) and half as high risk (*n* = 6; 50%) using the ROBIS tool (Appendix S3). It is worth noting that some risk of bias may have been attributed to incompatibility in select assessment domains due to the qualitative nature of the reviews included in this umbrella review.

Comparison of scoping reviews against the PRISMA‐ScR checklist demonstrated that all adhered to most of the essential reporting items. The main omission was failure to register a review protocol and/or to conduct and report results of critical appraisal. All scoping reviews cited the methodological framework outlined by Arksey and O'Malley^119^ whereby conduct of appraisal is reportedly not required. Together, this was interpreted as overall high quality for the scoping review included in this umbrella review.

## 4 DISCUSSION

### 4.1 Principal findings

This umbrella review explored when, how and to what extent CYP are involved in the conduct of health research, as well as the benefits, challenges and facilitators of involvement identified to date.

#### 4.1.1 Involvement must be flexible and accessible to suit CYP at all research stages

This umbrella review found that CYP (and/or their representatives) were involved in all stages of research, although to varying degrees. Involvement often occurred at multiple research stages within one study, the most prominent being research design and data collection. Involvement in research design was most often through verbal methods such as focus groups, interviews or advisory panels/co‐researcher roles, and more common when CYP were involved alongside representatives, perhaps due to the formal nature of these methods and complexity to aspects of research design. Inversely, involvement in data collection (contribution to the development of data collection tools and/or the conduct and implementation of data collection) was more common with CYP alone, through the aforementioned verbal methods, as well as creative visual methods like photography, video and/or drawing or active modes of expression (often used within a focus group or interview format). Regarding other stages of research, CYP (and/or their representatives) were least involved in data access and sharing, and funding decisions. This may not be surprising given the inherently technical aspects of these research processes. Furthermore, it is not always feasible to accommodate PPIE activities before obtaining funding.^2^, ^120^, ^121^ There exists a body of literature on the involvement of participants in research data governance, however this is largely focused on adult cohorts.^122^, ^123^, ^124^, ^125^ Consideration of with whom and how one's data are used and shared may also be of importance to CYP. Therefore, the involvement of CYP in data governance is an area that could benefit from greater attention.

The findings indicated that visual and active methods were more commonly used in studies involving CYP alone, rather than with their representatives. These methods tend to be less structured and more creative in nature, and therefore are more engaging for CYP and accommodate alternative modes of expression. Of the studies that reported on age of CYP, very few described involvement of only children ≤12. It is possible that this reflects barriers to planning and implementing projects involving younger children. These barriers may include, for example, restrictions on time, effort and funding, institutional and ethical requirements to suitably accommodate younger children,^9^, ^13^, ^95^, ^96^, ^99^, ^106^, ^107^, ^108^, ^112^, ^113^ as well as perceptions that younger children are less competent or capable of contributing value to research.^46^, ^96^, ^98^, ^102^, ^103^ Another confounding factor is how involvement of younger children is reported within the literature.^5^ Although studies often did not separate methodology across differing age groups, results suggested that active methods, such as role play or puppetry, were more likely to be used with children ≤ 12 than all CYP combined. Further research is needed to determine the extent to which methods need to be adapted to suit the age and developmental levels of those involved.

One emerging technique used for involvement of CYP was photovoice. Photovoice is a visual approach to research which allows an individual to document their lives and experiences through creative media.^73^ It helps to facilitate and remove barriers to communication and improve impact and translatability of findings through reflection.^126^ This method can be useful to support involvement of CYP who are unable to verbalise or directly express their experiences and perspectives.^22^, ^73^, ^110^ This is one example of how novel approaches can accommodate CYP with different needs and abilities. Bailey et al.^96^ also highlighted the importance of flexible and accessible methods to enable involvement of CYP with disabilities as partners in research, and Racine et al.^13^ discussed strategies to address system barriers for CYP with history of maltreatment.

#### 4.1.2 Researchers work with CYP at varying levels of involvement

This review found that researchers mostly ‘consulted’, ‘involved’ and ‘collaborated’ with CYP; however, very few ‘empowered’ CYP. Based on our modified IAP2 spectrum, this means that the feedback of CYP was sought and considered, that they worked alongside researchers in decision‐making, and that researchers shared responsibility with them. It is important to note that this umbrella review did not consider the lower ‘Inform’ level as meaningful involvement, and therefore did not comprehensively search and synthesise evidence of this level in the literature. We suspect that tokenistic involvement of CYP may be prevalent, particularly amongst older research when evidence and support to develop appropriate involvement approaches may have been more limited. In reality, tokenism can be unintentional and difficult to detect, in that despite best efforts, researchers might lack the resources and expertise to ensure that involvement is truly meaningful and influential.^21^, ^127^

Lower levels of involvement (‘consult’ and ‘involve’) are likely more prevalent due to barriers associated with higher levels (‘collaborate’ and ‘empower’). There may be lack of expertise in research teams, as well as financial and resource burdens associated with the conduct of successful and meaningful PPIE, particularly where collaboration opportunities are ongoing such as coresearcher roles.^72^, ^75^, ^116^, ^120^ In general, higher levels of involvement also require more power‐sharing, which can be challenging in any PPIE setting as it requires experts to relinquish some control of their research in favour of collaboration.^72^, ^103^, ^105^, ^120^ This often coincides with the unsubstantiated view that CYP do not have the maturity or competence to contribute value to research, particularly those <18 years of age.^117^, ^121^ Despite this, the literature demonstrates a rising commitment towards research that fosters true and equal partnership with CYP, with slightly elevated proportions of the higher levels of involvement (‘involve’, ‘collaborate’ and ‘empower’) for CYP alone. Research collaboration with CYP is increasingly more popular, such as the formation of advisory groups and committees guiding the decision‐making process for many of the research stages.^72^, ^75^, ^109^ Whilst still the minority, there were studies described in the reviews which demonstrated research empowerment with CYP.^9^, ^72^, ^99^, ^101^, ^103^, ^104^, ^107^ In most cases, this involved them leading their own research, or having an integrated role within the research project whereby youth‐led decisions and input was not overruled by adults.

Unfortunately, the level of involvement was only able to be inferred for just over one‐third of the studies included in the reviews. As such, it is possible that the findings described in this umbrella review may not accurately reflect the levels of involvement across all research.

#### 4.1.3 There is shared understanding of the benefits, challenges and facilitators for involvement of CYP

Almost all the reviews included in this umbrella review reported that involvement of CYP was beneficial in improving the relevancy of the research. It was also apparent that the contributions of CYP to the materials and methods of research, such as recruitment and data collection tools, can greatly benefit the completeness and quality of research results.^96^, ^99^, ^108^, ^128^ Facilitating meaningful involvement also helps to build trust between the community, researchers and policymakers, and together with the above benefits, enhances the dissemination and translatability of findings.^9^, ^104^, ^108^, ^128^

CYP also benefit from their involvement in the conduct of health research. The evidence suggests that there is an element of social empowerment that comes with having a say in matters that affect them.^9^, ^49^, ^104^, ^112^, ^114^, ^115^, ^121^ This seems of particular importance to CYP whom historically have not had a voice or opportunity to advocate for themselves in research and other adult‐powered settings.^13^, ^73^, ^121^ Furthermore, fair and successful involvement of CYP is often centred around giving back, such as through financial renumeration or attainment of skills and experience as appropriate for the age and developmental stage.^72^, ^75^, ^86^, ^96^, ^115^, ^116^

Despite substantial benefits, there were well‐recognised challenges to involvement of CYP in the conduct of health research such as those associated with time, costs, power imbalance, trust, meaningfulness and accessibility. However, the literature provided helpful suggestions to overcome hurdles and facilitate better involvement. The overarching recommendation was for researchers to plan adequately and be open minded towards involvement of CYP. Effort is required to ensure that involvement is accessible, engaging, and meaningful for both CYP and researchers. Involvement must not be tokenised; expectations should be managed from the beginning, and feedback provided regularly. Establishing respectful and power‐balanced relationships with CYP is one of the first steps towards successful involvement, as well as taking care to navigate conflicts with gatekeepers such as parents/caregivers.

Standards and best practices for involvement of CYP in research are emerging. There are some published guides for PPIE in research, including frameworks for reporting, monitoring and evaluating involvement^24^, ^89^, ^129^, ^130^, ^131^, ^132^ and a toolkit specifically for children's participation.^133^ However, most research studies have not systematically evaluated the impact of involvement and types of strategies employed. As such, recommended approaches in the literature are often based only on anecdotal evidence.^9^, ^63^, ^86^, ^104^, ^108^, ^112^, ^113^, ^114^, ^115^ It is important that researchers begin to incorporate evaluation into their PPIE planning so that outcomes can be assessed, and methods of involvement verified for their effectiveness.

## 5 STRENGTHS AND LIMITATIONS

To our knowledge, this is the first umbrella review on when, how and to what extent CYP are involved in the conduct of health research and fills a critical gap by providing a complete overview of the current landscape in this area. Our search strategy was designed to cover the breadth of the literature while maintaining sensitivity and specificity. However, there is a recognised difficulty in searching for relevant literature on this topic.^9^, ^95^, ^96^, ^101^, ^102^, ^107^, ^108^, ^112^, ^114^, ^115^ It is therefore plausible that included reviews may not have captured all relevant source articles. Furthermore, a lack of standardised terminology imposes a level of subjective interpretation on some findings.^104^, ^107^, ^108^ For example, terms ‘participation’, ‘involvement’ and ‘engagement’ were often used inconsistently and interchangeably across the literature, but can have vastly different meanings.^1^ This demonstrates a clear need for future research to consider consistency and intention in language used.

This umbrella review was successful in consolidating findings from the published literature and presenting common methodologies and approaches. We did not exclude any reviews based on the RoB assessment conducted due to the nonstandard reporting required for qualitative research on this topic. We therefore acknowledge the potential impact of bias on the findings of this umbrella review. It is also worth highlighting that many reviews and studies reported on the involvement of CYP across varying age groups and in conjunction with representatives. It was therefore difficult to ascertain which approaches were applicable for specific populations. Reviews describing representatives were included if they were involved alongside CYP. However, this umbrella review did not focus on involvement of representatives solely as informants for CYP. There were also some barriers in inferring the level of involvement as this was not consistently reported. The findings on this topic were therefore limited by the depth and accuracy of results presented by the included reviews.

## 6 CONCLUSION

This umbrella review identified consistency in the methods and approaches used, benefits and challenges reported and facilitators to support best practice involvement of CYP across research stages. It also highlighted important gaps in research processes and dissemination of findings. In particular, the need for consistency in reporting of PPIE, both in the terminology used and breadth of detail provided, to improve the quality, accessibility and useability of findings. Furthermore, approaches to involvement of CYP in research need to be better monitored and evaluated to demonstrate the impact on research and community outcomes.

## AUTHOR CONTRIBUTIONS

**Katherine A. Wyatt**: Investigation; writing—original draft; methodology; validation; visualization; writing—review and editing; formal analysis; project administration; data curation. **Jessica Bell**: Conceptualization; investigation; funding acquisition; methodology; writing—review and editing; project administration. **Jason Cooper**: Investigation; methodology; validation; visualization; writing—original draft; writing—review and editing; formal analysis. **Leanne Constable**: Conceptualization; investigation; methodology; validation; writing—review and editing; project administration. **William Siero**: Investigation; methodology; writing—review and editing; project administration. **Carla Pozo Jeria**: Conceptualization; investigation; methodology; writing—review and editing. **Simone Darling**: Conceptualization; investigation; funding acquisition; writing—review and editing. **Rachel Smith**: Conceptualization; investigation; funding acquisition; writing—review and editing. **Elizabeth K. Hughes**: Conceptualization; investigation; methodology; validation; writing— review and editing; project administration; supervision.

## CONFLICT OF INTEREST STATEMENT

The authors declare no conflict of interest.

## Supporting information

Supporting information.

Supporting information.

Supporting information.

## Data Availability

The data that support the findings of this study are available from the corresponding author upon reasonable request.

## References

[1] National Institute for Health and Care Research (NIHR) . Briefing notes for researchers—public involvement in NHS, Health and Social Care Research. 2021. Accessed February 9, 2024. https://www.nihr.ac.uk/documents/briefing-notes-for-researchers-public-involvement-in-nhs-health-and-social-care-research/27371

[2] Wilson ODL , Dieudonne L , Eustace J , et al. A Rapid Evidence Review of Young People's Involvement in Health Research. Wellcome; 2020.

[3] Bakhtiar A , Lang M , Shelley B , West M . Research with and by children: a systematic literature review. Rev Educ. 2023;11(1):e3384. 10.1002/rev3.3384

[4] Sun Y , Blewitt C , Edwards S , et al. Methods and ethics in qualitative research exploring young children's voice: a systematic review. Int J Qual Methods. 2023;22:16094069231152449. 10.1177/16094069231152449

[5] Freire K , Pope R , Jeffrey K , Andrews K , Nott M , Bowman T . Engaging with children and adolescents: a systematic review of participatory methods and approaches in research informing the development of health resources and interventions. Adolesc Res Rev. 2022;7(3):335‐354. 10.1007/s40894-022-00181-w

[6] Lundy L , McEvoy L . Freeman M , ed. Childhood, the United Nations Convention on the Rights of the Child, and Research: What Constitutes a ‘Rights‐Based’ Approach. Vol 14. Oxford University Press, 75‐91.

[7] Lundy L , McEvoy L . Children's rights and research processes: assisting children to (in)formed views. Childhood. 2011;19(1):129‐144. 10.1177/0907568211409078

[8] Lundy L , McEvoy L , Byrne B . Working with young children as co‐researchers: an approach informed by the United Nations Convention on the Rights of the Child. Early Educ Dev. 2011;22:714‐736. 10.1080/10409289.2011.596463

[9] McCabe E , Amarbayan M , Rabi S , et al. Youth engagement in mental health research: a systematic review. Health Expect. 2023;26(1):30‐50. 10.1111/hex.13650. 36385452 PMC9854331

[10] United Nations General Assembly Resolution . Convention on the rights of the child, A/RES/44/25. November 20, 1989. Accessed February 9, 2024. https://www.ohchr.org/en/instruments-mechanisms/instruments/convention-rights-child

[11] Montreuil M , Bogossian A , Laberge‐Perrault E , Racine E . A review of approaches, strategies and ethical considerations in participatory research with children. Int J Qual Methods. 2021;20:1609406920987962. 10.1177/1609406920987962

[12] New South Wales Government: Office of the Advocate for Children and Young People . Understanding and supporting children and young people's participation. 2015. Accessed February 9, 2024. https://www.acyp.nsw.gov.au/participation-resources/understanding-and-supporting-children-and-young-peoples-participation

[13] Racine N , Greer K , Dimitropoulos G , Collin‐Vézina D , Henderson JL , Madigan S . Youth engagement in child maltreatment research: gaps, barriers, and approaches. Child Abuse Negl. 2023;139:106127. 10.1016/j.chiabu.2023.106127 36907118

[14] Page MJ , McKenzie JE , Bossuyt PM , et al. The PRISMA 2020 statement: an updated guideline for reporting systematic reviews. BMJ. 2021;372:n71. 10.1136/bmj.n71 33782057 PMC8005924

[15] Pollock M , Fernandes R , Becker L , Pieper D , Hartling L . Chapter V: Overviews of reviews. In: Higgins JPT , Thomas J , Chandler J , eds. Cochrane Handbook for Systematic Reviews of Interventions Version 6.4 (Updated August 2023). Cochrane; 2023. https://training.cochrane.org/handbook

[16] Australian Institute of Health and Welfare . Australia's children. 2020. Accessed February 9, 2024. https://www.aihw.gov.au/getmedia/6af928d6-692e-4449-b915-cf2ca946982f/aihw-cws-69-print-report.pdf.aspx?inline=true

[17] World Health Organisation (WHO) . Adolescent Health. WHO; 2023. https://www.who.int/health-topics/adolescent-health#tab=tab_1

[18] United Nations General Assembly . *International Youth Year: Participation, Development, Peace*. Report A/36/215. June 19, 1981. Accessed February 9, 2024. https://undocs.org/en/A/36/215

[19] National Institute for Health and Care Research (NIHR): INVOLVE . What is public involvement in research? 2024. Accessed February 9, 2024. https://www.invo.org.uk/find-out-more/what-is-public-involvement-in-research-2/

[20] Clark J , Glasziou P , Del Mar C , Bannach‐Brown A , Stehlik P , Scott AM . A full systematic review was completed in 2 weeks using automation tools: a case study. J Clin Epidemiol. 2020;121:81‐90. 10.1016/j.jclinepi.2020.01.008 32004673

[21] Hahn DL , Hoffmann AE , Felzien M , LeMaster JW , Xu J , Fagnan LJ . Tokenism in patient engagement. Fam Pract. 2016;34(3):cmw097. 10.1093/fampra/cmw097 27660557

[22] Haijes HA , van Thiel GJMW . Participatory methods in pediatric participatory research: a systematic review. Pediatr Res. 2016;79(5):676‐683. 10.1038/pr.2015.279 26720607

[23] International Association for Public Participation . IAP2 spectrum of public participation. 2018. Accessed February 9, 2024. https://iap2.org.au/wp-content/uploads/2020/01/2018_IAP2_Spectrum.pdf

[24] Shier H , Berson I , Berson M . An analytical tool to help researchers develop partnerships with children and adolescents. In: Berson I , Berson M , Gray C , eds. Participatory Methodologies to Elevate Children's Voice and Agency. Information Age Publishing; 2019:295‐316.

[25] Pieper D , Antoine SL , Mathes T , Neugebauer EAM , Eikermann M . Systematic review finds overlapping reviews were not mentioned in every other overview. J Clin Epidemiol. 2014;67(4):368‐375. 10.1016/j.jclinepi.2013.11.007 24581293

[26] Whiting P , Savović J , Higgins JPT , et al. ROBIS: a new tool to assess risk of bias in systematic reviews was developed. J Clin Epidemiol. 2016;69:225‐234. 10.1016/j.jclinepi.2015.06.005 26092286 PMC4687950

[27] Tricco AC , Lillie E , Zarin W , et al. PRISMA extension for scoping reviews (PRISMA‐ScR): checklist and explanation. Ann Intern Med. 2018;169(7):467‐473. 10.7326/m18-0850 30178033

[28] Ibitoye BM , Garrett B , Ranger M , Stinson J . Conducting patient‐oriented research in low‐income and middle‐income countries: a scoping review. Patient. 2023;16(1):19‐29. 10.1007/s40271-022-00592-w 35869330

[29] de Laat J , Radner J , Holding P , et al. Measurement for change: reflections from innovators’ experiences with monitoring, evaluation, and learning systems for early childhood development. Front Public Health. 2023;11:1021790. 10.3389/fpubh.2023.1021790 37006525 PMC10060850

[30] Triplett NS , Woodard GS , Johnson C , et al. Stakeholder engagement to inform evidence‐based treatment implementation for children's mental health: a scoping review. Implement Sci Commun. 2022;3(1):82. 10.1186/s43058-022-00327-w 35906675 PMC9338493

[31] Rajamani G , Rodriguez Espinosa P , Rosas LG . Intersection of health informatics tools and community engagement in health‐related research to reduce health inequities: scoping review. J Particip Med. 2021;13(3):e30062. 10.2196/30062 34797214 PMC8663666

[32] Moore G , Wilding H , Gray K , Castle D . Participatory methods to engage health service users in the development of electronic health resources: systematic review. J Particip Med. 2019;11(1):e11474. 10.2196/11474 33055069 PMC7434099

[33] Levac L , Ronis S , Cowper‐Smith Y , Vaccarino O . A scoping review: the utility of participatory research approaches in psychology. J Community Psychol. 2019;47(8):1865‐1892. 10.1002/jcop.22231. 31441516 PMC6852237

[34] Fergusson D , Monfaredi Z , Pussegoda K , et al. The prevalence of patient engagement in published trials: a systematic review. Res Involv Engagem. 2018;4:17. 10.1186/s40900-018-0099-x 29796308 PMC5963039

[35] Menzies JC , Morris KP , Duncan HP , Marriott JF . Patient and public involvement in paediatric intensive care research: considerations, challenges and facilitating factors. Res Involv Engagem. 2016;2:32. 10.1186/s40900-016-0046-7 29507766 PMC5831882

[36] Brenner BL , Manice MP . Community engagement in children's environmental health research. Mt Sinai J Med. 2011;78(1):85‐97. 10.1002/msj.20231 21259265 PMC3086533

[37] Vaughn LM , Crosh C , Boyer K , Jenkins A . The possibility and promise of action research in pediatrics: a scoping review. Clin Pediatr.2023;62:99228221144838. 10.1177/00099228221144838 36625460

[38] Kaur P , Minhas R , Filler T , Torabi N . 148 A chair at the table: a scoping review of the participation of refugee adults and youth in healthcare research and policy design. Paediatr Child Health. 2021;26(suppl 1):e102. 10.1093/pch/pxab061.116

[39] Mandoh M , Redfern J , Mihrshahi S , Cheng HL , Phongsavan P , Partridge SR . How are adolescents engaged in obesity and chronic disease prevention policy and guideline development? A scoping review. Glob Health Res Policy. 2023;8(1):9. 10.1186/s41256-023-00294-2 36973812 PMC10041478

[40] Viksveen P , Bjønness SE , Cardenas NE , et al. User involvement in adolescents' mental healthcare: a systematic review. Eur Child Adolesc Psychiatry. 2022;31(11):1765‐1788. 10.1007/s00787-021-01818-2 34089383 PMC9666298

[41] Nguyen L , Jack S , Ketelaar M , Di Rezze B , Soper AK , Gorter JW . Understanding the essential components and experiences of youth with autism spectrum disorders in peer mentorship programmes during the transition to adulthood: a qualitative meta‐ethnography. Child Care Health Dev. 2020;46(6):667‐681. 10.1111/cch.12804 32840907

[42] Vujcich D , Thomas J , Crawford K , Ward J . Indigenous youth Peer‐Led health promotion in Canada, New Zealand, Australia, and the United States: a systematic review of the approaches, study designs, and effectiveness. Front Public Health. 2018;6:31. 10.3389/fpubh.2018.00031 29497608 PMC5818867

[43] Coyne I , O'Mathúna DP , Gibson F , Shields L , Leclercq E , Sheaf G . Interventions for promoting participation in shared decision‐making for children with cancer. Cochrane Database Syst Rev. 2016;2016(29):CD008970. 10.1002/14651858.CD008970.pub3 PMC6734120 27898175

[44] Yamaguchi S , Bentayeb N , Holtom A , et al. Participation of children and youth in mental health policymaking: a scoping review [part I]. Adm Policy Ment Health. 2023;50(1):58‐83. 10.1007/s10488-022-01223-0 36357819

[45] Anderson AJ . A qualitative systematic review of youth participatory action research implementation in U.S. high schools. Am J Community Psychol. 2020;65(1‐2):242‐257. 10.1002/ajcp.12389 31489643

[46] Teela L , Verhagen LE , van Oers HA , et al. Pediatric patient engagement in clinical care, research and intervention development: a scoping review. J Patient Rep Outcomes. 2023;7(1):32. 10.1186/s41687-023-00566-y 36988738 PMC10060502

[47] Cwintal M , Ranjbar H , Bandamiri P , Guadagno E , Osmanlliu E , Poenaru D . A rapid review for developing a co‐design framework for a pediatric surgical communication application. J Pediatr Surg. 2023;58:879‐890. 10.1016/j.jpedsurg.2023.01.030 36805140

[48] Gurung G , Richardson A , Wyeth E , Edmonds L , Derrett S . Child/youth, family and public engagement in paediatric services in high‐income countries: a systematic scoping review. Health Expect. 2020;23(2):261‐273. 10.1111/hex.13017 31981295 PMC7104655

[49] Anyon Y , Bender K , Kennedy H , Dechants J . A systematic review of youth participatory action research (YPAR) in the United States: methodologies, youth outcomes, and future directions. Health Educ Behav. 2018;45(6):865‐878. 10.1177/1090198118769357 29749267

[50] Ackerman TF . The ethics of drug research in children. Paediatr Drugs. 2001;3(1):29‐41. 11220403 10.2165/00128072-200103010-00003

[51] Povey J , Raphiphatthana B , Torok M , et al. An emerging framework for digital mental health design with Indigenous young people: a scoping review of the involvement of Indigenous young people in the design and evaluation of digital mental health interventions. Syst Rev. 2023;12(1):108. 10.1186/s13643-023-02262-w 37393283 PMC10314399

[52] Hurd NM , Brence M , Armstrong C . Youth advancing anti‐racism in the 2020s. Curr Opin Psychol. 2023;52:101612. 10.1016/j.copsyc.2023.101612 37354570

[53] Napier‐Raman S , Hossain SZ , Lee MJ , Mpofu E , Liamputtong P , Dune T . Migrant and refugee youth perspectives on sexual and reproductive health and rights in Australia: a systematic review. Sex Health. 2023;20(1):35‐48. 10.1071/SH22081 36455882

[54] Adair B , Ullenhag A , Rosenbaum P , Granlund M , Keen D , Imms C . Abstract papers. Dev Med Child Neurol. 2018;60(suppl 1):4‐60. 10.1111/dmcn.13665 30022476

[55] Broome ME . Consent (assent) for research with pediatric patients. Semin Oncol Nurs. 1999;15(2):96‐103. 10222509 10.1016/s0749-2081(99)80067-9

[56] Clavering EK , McLaughlin J . Children's participation in health research: from objects to agents. Child Care Health Dev. 2010;36(5):603‐611. 10.1111/j.1365-2214.2010.01094.x 20533922

[57] D'aprano A , Carmody S , Manahan E , Savaglio M , Galvin E , Skouteris H . A rapid review of implementation frameworks underpinning aboriginal and Torres Strait Islander children's health and social care programs. Aust N Z J Public Health. 2023;47(3):100063. 10.1016/j.anzjph.2023.100063 37267813

[58] Khanom S , McBeth J , Briggs M , Bakir E , McDonagh J . Adolescents' experiences of fluctuating pain in musculoskeletal disorders: a qualitative systematic review and thematic synthesis. Rheumatology. 2019;58(suppl 4):IV18. 10.1093/rheumatology/kez416.003 PMC7532580 33008357

[59] Macrae D . Conducting clinical trials in pediatrics. Crit Care Med. 2009;37(suppl 1):S136‐S139. 10.1097/CCM.0b013e318192101f 19104213

[60] Miller VA , Drotar D , Kodish E . Children's competence for assent and consent: a review of empirical findings. Ethics Behav. 2004;14(3):255‐295. 15875339 10.1207/s15327019eb1403_3

[61] Namisango E , Bristowe K , Allsop MJ , et al. Symptoms and concerns among children and young people with life‐limiting and life‐threatening conditions: a systematic review highlighting meaningful health outcomes. Patient. 2019;12(1):15‐55. 10.1007/s40271-018-0333-5 30361884

[62] Orlowski SK , Lawn S , Venning A , et al. Participatory research as one piece of the puzzle: a systematic review of consumer involvement in design of technology‐based youth mental health and well‐being interventions. JMIR Hum Factors. 2015;2(2):e12. 10.2196/humanfactors.4361 27025279 PMC4797690

[63] Ozer EJ , Abraczinskas M , Duarte C , et al. Youth participatory approaches and health equity: conceptualization and integrative review. Am J Community Psychol. 2020;66(3‐4):267‐278. 10.1002/ajcp.12451 32969506

[64] Priest N , Mackean T , Waters E , Davis E , Riggs E . Indigenous child health research: a critical analysis of Australian studies. Aust N Z J Public Health. 2009;33(1):55‐63. 10.1111/j.1753-6405.2009.00339.x 19236360

[65] Scherer DG , Annett RD , Brody JL . Ethical issues in adolescent and parent informed consent for pediatric asthma research participation. J Asthma. 2007;44(7):489‐496. 17885849 10.1080/02770900701247137

[66] Van Goidsenhoven L , De Schauwer E . Relational ethics, informed consent, and informed assent in participatory research with children with complex communication needs. Dev Med Child Neurol. 2022;64(11):1323‐1329. 10.1111/dmcn.15297 35665498

[67] Wilkins KL , Woodgate RL . A review of qualitative research on the childhood cancer experience from the perspective of siblings: a need to give them a voice. J Pediatr Oncol Nurs. 2005;22(6):305‐319. 16216893 10.1177/1043454205278035

[68] Kasperbauer TJ , Halverson C . Adolescent assent and reconsent for biobanking: recent developments and emerging ethical issues. Front Med. 2021;8:686264. 10.3389/fmed.2021.686264 PMC8301072 34307413

[69] Anonymous . Ethical considerations in research with socially identifiable populations. Pediatrics. 2004;113(1 I):148‐151. 10.1542/peds.113.1.148 14702468

[70] Burack S , Wiedenman EM , Ward M , Kaufman L , Shato T , Hunleth J . Abstract PO‐120: need for more sociodemographic data in qualitative childhood cancer research: findings from a scoping review. Cancer Epidemiol Biomarkers Prevent. 2022;31(1 suppl):PO‐120. 10.1158/1538-7755.DISP21-PO-120

[71] Chagnon K , Montreuil M . Partnering with children in healthcare: no partnership without trust. BMJ Open. 2021;11(suppl 1):A2‐A3. 10.1136/bmjopen-2021-QHRN.6

[72] Chan M , Scott SD , Campbell A , Elliott SA , Brooks H , Hartling L . Research‐ and health‐related youth advisory groups in Canada: an environmental scan with stakeholder interviews. Health Expect. 2021;24(5):1763‐1779. 10.1111/hex.13316 34288282 PMC8483214

[73] D'Amico M , Denov M , Khan F , Linds W , Akesson B . Research as intervention? Exploring the health and well‐being of children and youth facing global adversity through participatory visual methods. Global Public Health. 2016;11(5‐6):528‐545. 10.1080/17441692.2016.1165719 27043374

[74] Damne E , Chaplin J . 63rd Annual Conference of Indian Society of Hematology & Blood Transfusion (ISHBT) November 2022. Indian J Hematol Blood Transfus. 2022;38(suppl 2):S92. 10.1007/s12288-022-01605-2

[75] Del Gaizo V , Kohlheim M . Patient engagement in pediatric rheumatology research. Rheumatic Dis Clin North Am. 2022;48(1):1‐13. 10.1016/j.rdc.2021.09.013 34798941

[76] Doyle AM , Dziva Chikwari C , Majozi N , et al. Adolescent health series: engagement with young people as partners in health research: four case studies from Sub‐Saharan Africa. Trop Med Int Health. 2022;27(1):2‐12. 10.1111/tmi.13702 34861086

[77] Gaillard S , Malik S , Preston J , et al. Involving children and young people in clinical research through the forum of a European Young Persons' Advisory Group: needs and challenges. Fundam Clin Pharmacol. 2018;32(4):357‐362. 10.1111/fcp.12360 29457267

[78] Gavine A , Aleman‐Diaz AY , Currie C , Garcia‐Moya I , Humphris G , Morgan A . 9th excellence in Pediatrics Conference—2017 Book of Abstracts. Cogent Med. 2017;4(1):1408251. 10.1080/2331205X.2017.1408251

[79] Joffe S , Fernandez CV , Pentz RD , et al. Involving children with cancer in decision‐making about research participation. J Pediatr. 2006;149(6):862‐868.e1. 17137908 10.1016/j.jpeds.2006.08.027

[80] Kowalewska B , Drozdz W , Kowalewski L , Skibicka M , Gizinski L , Ceannt R . E‐poster viewing. Eur Psychiatry. 2019;56(suppl):S405. 10.1016/j.eurpsy.2019.01.002

[81] Leadbeater B , Marshall A , Banister E . Building strengths through practice‐research‐policy collaborations. Child Adolesc Psychiatr Clin N Am. 2007;16(2):515‐532. 17349521 10.1016/j.chc.2006.11.004

[82] Nelson KM . Designing healthier communities through the input of children. J Public Health Manag Pract. 2008;14(3):266‐271. 10.1097/01.PHH.0000316485.49888.f6 18408551

[83] Oliveras C , Cluver L , Bernays S , Armstrong A . Nothing about us without RIGHTS—meaningful engagement of children and youth: from research prioritization to clinical trials, implementation science, and policy. J Acquir Immune Defic Syndr. 2018;78(suppl 1):S27‐S31. 10.1097/QAI.0000000000001746 29994917 PMC6075896

[84] Phillips B , Davies HT , Preston J , Stones SR . Framework to help design and review research involving children. Arch Dis Child. 2019;104(6):601‐604. 10.1136/archdischild-2018-315119 31101643

[85] Powers JL , Tiffany JS . Engaging youth in participatory research and evaluation. J Public Health Manag Pract. 2006;12(suppl 6):S79‐S87. 10.1097/00124784-200611001-00015 17035908

[86] Prebeg M , Patton M , Desai R , et al. From participants to partners: reconceptualising authentic patient engagement roles in youth mental health research. Lancet Psychiatry. 2023;10(2):139‐145. 10.1016/S2215-0366(22)00377-7 36502816

[87] Preston J , Nafria B , Ohmer A , et al. Developing a more tailored approach to patient and public involvement with children and families in pediatric clinical research: lessons learned. Ther Innov Regul Sci. 2022;56(6):948‐963. 10.1007/s43441-022-00382-4 35182389 PMC8857393

[88] Saberi P , Campbell CK , Venegas M , Dubé K . Time to engage young people in HIV cure research. AIDS Res Hum Retroviruses. 2022;38(1):2‐4. 10.1089/aid.2020.0268 33677996 PMC8785756

[89] Surko M , Lawson HA , Gaffney S , Claiborne N . Targeting evaluations of youth development‐oriented community partnerships. J Public Health Manag Pract. 2006;12(suppl 6):S95‐S107. 10.1097/00124784-200611001-00017 17035910

[90] Teela L , Verhagen L , Grootenhuis M , Haverman L . 25th annual conference of the international society for quality of life research. Qual Life Res. 2018;27(suppl 1):1‐190. 10.1007/s11136-018-1946-9 30196342

[91] Thornton H . Information and involvement. Health Expect. 2001;4(1):71‐74. 10.1046/j.1369-6513.2001.00129.x 11286601 PMC5060040

[92] Vanska N , Sipari S , Lehtonen K . Posters. Dev Med Child Neurol. 2022;64(suppl 3):54‐100. 10.1111/dmcn.15215

[93] Wall MA , Jenney A , Walsh M . Conducting evaluation research with children exposed to violence: how technological innovations in methodologies and data collection May enhance the process. Child Abuse Negl. 2018;85:202‐208. 10.1016/j.chiabu.2018.01.007 29366597

[94] Wray J , Terrell K , Kelly P , Chesters H , Gibson F , Oldham G . Use of patient‐reported experience measures (PREMs) and patient‐reported outcome measures (PROMs) in routine hospital care of children and young people: a scoping literature review. Arch Dis Child. 2023;108(suppl 1):A25‐A26. 10.1136/archdischild-2023-gosh.67

[95] Ali AZ , Wright B , Curran JA , Newton AS . Review: patient engagement in child, adolescent, and youth mental health care research—a scoping review. Child Adolesc Mental Health. 2023;28:524‐535. 10.1111/camh.12615 36494910

[96] Bailey S , Boddy K , Briscoe S , Morris C . Involving disabled children and young people as partners in research: a systematic review. Child Care Health Dev. 2015;41(4):505‐514. 10.1111/cch.12197 25323964

[97] Branquinho C , Tomé G , Grothausen T , Gaspar de Matos M . Community‐based youth participatory action research studies with a focus on youth health and well‐being: a systematic review. J Community Psychol. 2020;48(5):1301‐1315. 10.1002/jcop.22320 31985839

[98] Dubois AC , Lahaye M , Aujoulat I . From research ‘on’ to research ‘with’ children about their family lives: a scoping review of ethical and methodological challenges. Child Care Health Dev. 2022;48(2):203‐216. 10.1111/cch.12937 34859480

[99] Flynn R , Walton S , Scott SD . Engaging children and families in pediatric health research: a scoping review. Res Involv Engagem. 2019;5:32. 10.1186/s40900-019-0168-9 31700676 PMC6827239

[100] Fountain S , Hale R , Spencer N , Morgan J , James L , Stewart MK . A 10‐Year systematic review of photovoice projects with youth in the United States. Health Promot Pract. 2021;22(6):767‐777. 10.1177/15248399211019978 34269073

[101] Gibbs L , Kornbluh M , Marinkovic K , Bell S , Ozer EJ . Using technology to scale up youth‐led participatory action research: a systematic review. J Adolesc Health. 2020;67(2S):S14‐S23. 10.1016/j.jadohealth.2019.10.019 32718510

[102] Hunleth JM , Spray JS , Meehan C , Lang CW , Njelesani J . What is the state of children's participation in qualitative research on health interventions?: a scoping study. BMC Pediatr. 2022;22(1):328. 10.1186/s12887-022-03391-2 35659206 PMC9166159

[103] Larsson I , Staland‐Nyman C , Svedberg P , Nygren JM , Carlsson IM . Children and young people's participation in developing interventions in health and well‐being: a scoping review. BMC Health Serv Res. 2018;18(28):507. 10.1186/s12913-018-3219-2 29954392 PMC6027768

[104] Mandoh M , Redfern J , Mihrshahi S , Cheng HL , Phongsavan P , Partridge SR . Shifting from tokenism to meaningful adolescent participation in research for obesity prevention: a systematic scoping review. Front Public Health. 2021;9:789535. 10.3389/fpubh.2021.789535 35004591 PMC8734426

[105] McNeill M , Noyek S , Engeda E , Fayed N . Assessing the engagement of children and families in selecting patient‐reported outcomes (PROs) and developing their measures: a systematic review. Qual Life Res. 2021;30(4):983‐995. 10.1007/s11136-020-02690-4 33156433

[106] Nathan S , Hodgins M , Wirth J , Ramirez J , Walker N , Cullen P . The use of arts‐based methodologies and methods with young people with complex psychosocial needs: a systematic narrative review. Health Expect. 2023;26(2):795‐805. 10.1111/hex.13705 36628644 PMC10010092

[107] Nortvedt L , Olsen CF , Sjølie H . Young peoples' involvement in welfare service development—is voice enough?—a thematic synthesis of qualitative studies. Health Expect. 2022;25(4):1464‐1477. 10.1111/hex.13485 35318770 PMC9327858

[108] Rouncefield‐Swales A , Harris J , Carter B , Bray L , Bewley T , Martin R . Children and young people's contributions to public involvement and engagement activities in health‐related research: a scoping review. PLoS One. 2021;16(6):e0252774. 10.1371/journal.pone.0252774 34106978 PMC8189547

[109] Sellars E , Pavarini G , Michelson D , Creswell C , Fazel M . Young people's advisory groups in health research: scoping review and mapping of practices. Arch Dis Child. 2021;106(7):698‐704. 10.1136/archdischild-2020-320452 33208398

[110] Sposito AMP , Sparapani VC , Pfeifer LI , Lima RAG , Nascimento LC . Estratégias lúdicas de coleta de dados com crianças com câncer: revisão integrativa. Revista Gaúcha de Enfermagem. 2013;34(3):187‐195. 10.1590/s1983-14472013000300024 24344602

[111] Thomas C , Cockcroft E , Jenkins G , Liabo K . Working with children and young people in research: supportive practices and pathways to impact. J Child Health Care. Forthcoming 2024. 10.1177/13674935231171451 PMC11874604 37186542

[112] Valdez ES , Skobic I , Valdez L , et al. Youth participatory action research for youth substance use prevention: a systematic review. Subst Use Misuse. 2020;55(2):314‐328. 10.1080/10826084.2019.1668014 31596160 PMC8034545

[113] van Schelven F , Boeije H , Mariën V , Rademakers J . Patient and public involvement of young people with a chronic condition in projects in health and social care: a scoping review. Health Expect. 2020;23(4):789‐801. 10.1111/hex.13069 32372423 PMC7495073

[114] Vanderhout SM , Bhalla M , Van A , et al. The impact of patient and family engagement in child health research: a scoping review. J Pediatr. 2023;253:115‐128. 10.1016/j.jpeds.2022.09.030 36179891

[115] Vaughn LM , Wagner E , Jacquez F . A review of community‐based participatory research in child health. Am J Maternal/Child Nurs. 2013;38(1):48‐53. 10.1097/NMC.0b013e31826591a3 23232779

[116] Jørgensen CR . Children's involvement in research—a review and comparison with service user involvement in health and social care. Soc Sci. 2019;8(5):149.

[117] Bradbury‐Jones C , Taylor J . Engaging with children as co‐researchers: challenges,counter‐challenges and solutions. Int J Soc Res Methodol. 2015;18(2):161‐173. 10.1080/13645579.2013.864589

[118] Ozer EJ . Youth‐Led participatory action research: developmental and equity. Adv Child Dev Behav. 2016;50:189‐207. 10.1016/bs.acdb.2015.11.006 26956074

[119] Arksey H , O'Malley L . Scoping studies: towards a methodological framework. Int J Soc Res Methodol. 2005;8(1):19‐32. 10.1080/1364557032000119616

[120] McLaughlin H . Involving young service users as co‐researchers: possibilities, benefits and costs. Br J Soc Work. 2005;36(8):1395‐1410. 10.1093/bjsw/bch420

[121] Alderson P . Research by children. Int J Soc Res Methodol. 2001;4(2):139‐153. 10.1080/13645570120003

[122] Kaye J , Terry SF , Juengst E , et al. Including all voices in international data‐sharing governance. Hum Genomics. 2018;12(1):13. 10.1186/s40246-018-0143-9 29514717 PMC5842530

[123] Coathup V , Hamakawa N , Finlay T , Bell J , Kaye J , Kato K . Participant‐Centric initiatives and medical research: scoping review protocol. JMIR Res Protoc. 2017;6(12):e245. 10.2196/resprot.7407 29233800 PMC5743923

[124] Hunter KG , Laurie GT . Involving publics in biobank governance: moving beyond existing approaches. In: Mullen C , Widdows H , eds. The Governance of Genetic Information: Who Decides? Cambridge Law, Medicine and Ethics. Cambridge University Press; 2009:151‐177.

[125] Erikainen S , Friesen P , Rand L , et al. Public involvement in the governance of population‐level biomedical research: unresolved questions and future directions. J Med Ethics. 2021;47(7):522‐525. 10.1136/medethics-2020-106530 33023977

[126] Wang C , Burris MA . Photovoice: concept, methodology, and use for participatory needs assessment. Health Educ Behav. 1997;24(3):369‐387. 10.1177/109019819702400309 9158980

[127] Romsland GI , Milosavljevic KL , Andreassen TA . Facilitating non‐tokenistic user involvement in research. Res Involv Engagem. 2019;5(1):18. 10.1186/s40900-019-0153-3 31183162 PMC6551868

[128] Preston J , Stones SR , Davies H , Phillips B . How to involve children and young people in what is, after all, their research. Arch Dis Child. 2019;104(5):494‐500. 10.1136/archdischild-2018-315118 31000534

[129] Green T , Bonner A , Teleni L , et al. Use and reporting of experience‐based codesign studies in the healthcare setting: a systematic review. BMJ Qual Saf. 2020;29(1):64‐76. 10.1136/bmjqs-2019-009570 31548278

[130] Staniszewska S , Brett J , Simera I , et al. GRIPP2 reporting checklists: tools to improve reporting of patient and public involvement in research. Res Involv Engagem. 2017;3(1):13. 10.1186/s40900-017-0062-2 29062538 PMC5611595

[131] Morrow E , Ross F , Grocott P , Bennett J . A model and measure for quality service user involvement in health research. Int J Consum Stud. 2010;34:532‐539. 10.1111/j.1470-6431.2010.00901.x

[132] Collins M , Long R , Page A , Popay J , Lobban F . Using the Public Involvement Impact Assessment Framework to assess the impact of public involvement in a mental health research context: a reflective case study. Health Expect. 2018;21(6):950‐963. 10.1111/hex.12688 29696740 PMC6250886

[133] Lansdown G , O'Kane C . A Toolkit for Monitoring and Evaluating Children's Participation. Save the Children; 2014.

