# Supplementary material for: Involvement of children and young people in the conduct of health research: A rapid umbrella review
Source: Health Expect. 2024 Jun 6;27(3):e14081. doi: 10.1111/hex.14081 (PMC11156690; doi:10.1111/hex.14081)
Supplement: Supplementary file 1 — Supporting information. [file HEX-27-e14081-s001.docx]

**Appendix 1:** Search query for reviews and meta-analyses

**Ovid MEDLINE(R) ALL** <Conception (1946) to 29 August 2023>

1 Patient Participation/

2 Stakeholder Participation/

3 exp Community Participation/

4 exp Community-Based Participatory Research/

5 (CBPR or participatory-research* or participatory-method* or participatory-approach* or participatory-design or participatory-model*).tw,kf.

6 Advisory Committees/

7 (advisory-group* or advisory-panel* or advisory-board? or advisory-committee* or focus-group* or expert-group* or ambassador*).tw,kf.

8 (co-produc* or coproduc* or co-design* or codesign* or co-research* or coresearch* or co-develop* or codevelop* or co-creation* or cocreation*).tw,kf.

9 (patient* or public or communit* or user* or stakeholder* or consumer* or client* or citizen* or participant*).tw,kf.

10 ((participat* or involv* or consult* or collaborat* or engage* or inform? or informed or informing or contribute or contribution or contributed or contributing or guide? or guiding or guided or guidance* or impact* or input or influenc* or advis* or advocat* or "led" or partner*) adj5 ((research or study or studies or project?) adj3 (design* or model* or program? or programme? or stag* or approach or approaches or process or processes or procedure* or activit* or deliver* or decision? or decision-making or develop* or implement* or execut* or plan? or planning or governance or polic*))).tw,kf.

11 ((participat* or involv* or consult* or collaborat* or engage* or inform? or informed or informing or contribute or contribution or contributed or contributing or guide? or guiding or guided or guidance* or impact* or input or influenc* or advis* or advocat* or "led" or partner*) adj5 ((data or sample? or result? or finding?) adj3 (collect* or analys* or analyz* or disseminat* or "use" or usage or share* or sharing or linkage? or linking or governance or polic*))).tw,kf.

12 1 or 2 or 3 or 4 or 5 or 6 or 7 or 8 or 9

13 10 or 11

14 12 and 13

15 (Patient-and-public-involvement or Patient-and-public-engagement or Public-and-patient-involvement or Public-and-patient-engagement or Patient -public-involvement or Patient-public-engagement or Public-patient-involvement or Public-patient-engagement or Youth-Advisory-Group* or Young-People's-Advisory-Group* or Young-Person's-Advisory-Group* or Young-People's-Action-Group* or Youth-Participatory-Action-Research or Youth-Led-Participatory-Action-Research or YPAG or YPAGs or PPIE or YPAR).tw,kf.

16 14 or 15

17 (health* or biomedical or medic* or clinic*).tw,kf,hw,jw.

18 (newborn* or new-born* or baby or babies or neonat* or neo-nat* or infan* or toddler* or pre-schooler* or preschooler* or kinder or kinders or kindergarten* or kinder-aged or boy or boys or girl or girls or child or children or childhood or youngster* or kid or kids or preadolescen* or pre-puberty or prepuberty or pre-pubescen* or prepubescen* or adolescen* or pediatric* or paediatric* or youth or youths or teen or teens or teenage* or juvenile* or minor or minors or underage* or under-age* or school-age* or schoolage* or schoolchild* or schoolgirl* or schoolboy* or young-adult* or young-people* or young-person* or emerging-adult* or emerging-people* or emerging-person* or AYA or AYAs).tw,kf,hw.

19 (systematic-review or rapid-review or meta-analysis or meta-synthesis or meta-review or literature-review or scoping-review or umbrella-review or narrative-review).tw,kf,hw. or Systematic Review/ or Review/

20 16 and 17 and 18 and 19

**PubMed (NLM)** <Conception to 29 August 2023>

Filters applied: Meta-Analysis, Review, Systematic Review

1 "Patient-and-public-involvement"[Title/Abstract] OR "Patient-and-public-engagement"[Title/Abstract] OR "Public-and-patient-involvement"[Title/Abstract] OR "Public-and-patient-engagement"[Title/Abstract] OR "Patient-public-involvement"[Title/Abstract] OR "Patient-public-engagement"[Title/Abstract] OR "Public-patient-involvement"[Title/Abstract] OR "Public-patient-engagement"[Title/Abstract] OR "Youth-Advisory-Group*"[Title/Abstract] OR "Young-People's-Advisory-Group*"[Title/Abstract] OR "Young-Person's-Advisory-Group*"[Title/Abstract] OR "Young-People's-Action-Group*"[Title/Abstract] OR "Youth-Participatory-Action-Research"[Title/Abstract] OR "Youth-Led-Participatory-Action-Research"[Title/Abstract] OR "YPAG"[Title/Abstract] OR "YPAGs"[Title/Abstract] OR "CBPR"[Title/Abstract] OR "PPIE"[Title/Abstract] OR "YPAR"[Title/Abstract]

2 "advisory-group*"[Title/Abstract] OR "advisory-panel*"[Title/Abstract] OR "advisory-board*"[Title/Abstract] OR "advisory-committee*"[Title/Abstract] OR "focus-group*"[Title/Abstract] OR "expert-group*"[Title/Abstract] OR "ambassador*"[Title/Abstract] OR "participatory-research*"[Title/Abstract] OR "participatory-method*"[Title/Abstract] OR "participatory-approach*"[Title/Abstract] OR "participatory-design"[Title/Abstract] OR "participatory-model*"[Title/Abstract] OR "co-produc*"[Title/Abstract] OR "coproduc*"[Title/Abstract] OR "co-design*"[Title/Abstract] OR "codesign*"[Title/Abstract] OR "co-research*"[Title/Abstract] OR "coresearch*"[Title/Abstract] OR "co-develop*"[Title/Abstract] OR "codevelop*"[Title/Abstract] OR "co-creation*"[Title/Abstract] OR "cocreation*"[Title/Abstract] OR "patient*"[Title/Abstract] OR "public"[Title/Abstract] OR "communit*"[Title/Abstract] OR "user*"[Title/Abstract] OR "stakeholder*"[Title/Abstract] OR "consumer*"[Title/Abstract] OR "client*"[Title/Abstract] OR "citizen*"[Title/Abstract] OR "participant*"[Title/Abstract]

3 "participatory research design"[Title/Abstract:~4] OR "participatory research process"[Title/Abstract:~4] OR "participatory research decision"[Title/Abstract:~4] OR "participatory research development"[Title/Abstract:~4] OR "participatory research approaches"[Title/Abstract:~4] OR "participatory research stages"[Title/Abstract:~4] OR "participation research design"[Title/Abstract:~4] OR "participation research process"[Title/Abstract:~4] OR "participation research decision"[Title/Abstract:~4] OR "participation research development"[Title/Abstract:~4] OR "participation research approaches"[Title/Abstract:~4] OR "participation research stages"[Title/Abstract:~4] OR "participate research design"[Title/Abstract:~4] OR "participate research process"[Title/Abstract:~4] OR "participate research decision"[Title/Abstract:~4] OR "participate research development"[Title/Abstract:~4] OR "participate research approaches"[Title/Abstract:~4] OR "participate research stages"[Title/Abstract:~4]

4 "involvement research design"[Title/Abstract:~4] OR "involvement research process"[Title/Abstract:~4] OR "involvement research decision"[Title/Abstract:~4] OR "involvement research development"[Title/Abstract:~4] OR "involvement research approaches"[Title/Abstract:~4] OR "involvement research stages"[Title/Abstract:~4] OR "involved research design"[Title/Abstract:~4] OR "involved research process"[Title/Abstract:~4] OR "involved research decision"[Title/Abstract:~4] OR "involved research development"[Title/Abstract:~4] OR "involved research approaches"[Title/Abstract:~4] OR "involved research stages"[Title/Abstract:~4]

5 "engagement research design"[Title/Abstract:~4] OR "engagement research process"[Title/Abstract:~4] OR "engagement research decision"[Title/Abstract:~4] OR "engagement research development"[Title/Abstract:~4] OR "engagement research approaches"[Title/Abstract:~4] OR "engagement research stages"[Title/Abstract:~4] OR "engaged research design"[Title/Abstract:~4] OR "engaged research process"[Title/Abstract:~4] OR "engaged research decision"[Title/Abstract:~4] OR "engaged research development"[Title/Abstract:~4] OR "engaged research approaches"[Title/Abstract:~4] OR "engaged research stages"[Title/Abstract:~4] OR "engagement study design"[Title/Abstract:~4] OR "engagement studies design"[Title/Abstract:~4]

6 "informed research design"[Title/Abstract:~4] OR "informed research process"[Title/Abstract:~4] OR "informed research decision"[Title/Abstract:~4] OR "informed research development"[Title/Abstract:~4] OR "informed research approaches"[Title/Abstract:~4] OR "informed research stages"[Title/Abstract:~4] OR "impact research design"[Title/Abstract:~4] OR "impact research process"[Title/Abstract:~4] OR "impact research decision"[Title/Abstract:~4] OR "impact research development"[Title/Abstract:~4] OR "impact research approaches"[Title/Abstract:~4] OR "impact research stages"[Title/Abstract:~4] OR "partners research design"[Title/Abstract:~4] OR "partners research process"[Title/Abstract:~4] OR "partners research decision"[Title/Abstract:~4] OR "partners research development"[Title/Abstract:~4] OR "partners research approaches"[Title/Abstract:~4] OR "partners research stages"[Title/Abstract:~4]

7 "influence research design"[Title/Abstract:~4] OR "influence research process"[Title/Abstract:~4] OR "influence research decision"[Title/Abstract:~4] OR "influence research development"[Title/Abstract:~4] OR "influence research approaches"[Title/Abstract:~4] OR "influence research stages"[Title/Abstract:~4] OR "influence study development"[Title/Abstract:~4] OR "influence studies development"[Title/Abstract:~4] OR "influencing research design"[Title/Abstract:~4] OR "influencing research process"[Title/Abstract:~4] OR "influencing research decision"[Title/Abstract:~4] OR "influencing research development"[Title/Abstract:~4] OR "influencing research approaches"[Title/Abstract:~4] OR "influencing research stages"[Title/Abstract:~4]

8 "contribute research design"[Title/Abstract:~4] OR "contribute research process"[Title/Abstract:~4] OR "contribute research decision"[Title/Abstract:~4] OR "contribute research development"[Title/Abstract:~4] OR "contribute research approaches"[Title/Abstract:~4] OR "contribute research stages"[Title/Abstract:~4] OR "contribution research design"[Title/Abstract:~4] OR "contribution research process"[Title/Abstract:~4] OR "contribution research decision"[Title/Abstract:~4] OR "contribution research development"[Title/Abstract:~4] OR "contribution research approaches"[Title/Abstract:~4] OR "contribution research stages"[Title/Abstract:~4]

9 #2 AND (#3 OR #4 OR #5 OR #6 OR #7 OR #8)

10 #1 OR #9

11 "health*"[Title/Abstract] OR "biomedical"[Title/Abstract] OR "medic*"[Title/Abstract] OR "clinic*"[Title/Abstract] OR "trial*"[Title/Abstract] OR "hospital*"[Title/Abstract]

12 "newborn*"[Title/Abstract] OR "new-born*"[Title/Abstract] OR "baby"[Title/Abstract] OR "babies"[Title/Abstract] OR "neonat*"[Title/Abstract] OR "neo-nat*"[Title/Abstract] OR "infan*"[Title/Abstract] OR "toddler*"[Title/Abstract] OR "pre-schooler*"[Title/Abstract] OR "preschooler*"[Title/Abstract] OR "kinder"[Title/Abstract] OR "kinders"[Title/Abstract] OR "kindergarten*"[Title/Abstract] OR "kinder-aged"[Title/Abstract] OR "boy"[Title/Abstract] OR "boys"[Title/Abstract] OR "girl"[Title/Abstract] OR "girls"[Title/Abstract] OR "child"[Title/Abstract] OR "children"[Title/Abstract] OR "childhood"[Title/Abstract] OR "youngster*"[Title/Abstract] OR "kid"[Title/Abstract] OR "kids"[Title/Abstract] OR "preadolescen*"[Title/Abstract] OR "pre-puberty"[Title/Abstract] OR "prepuberty"[Title/Abstract] OR "pre-pubescen*"[Title/Abstract] OR "prepubescen*"[Title/Abstract] OR "adolescen*"[Title/Abstract] OR "pediatric*"[Title/Abstract] OR "paediatric*"[Title/Abstract] OR "youth"[Title/Abstract] OR "youths"[Title/Abstract] OR "teen"[Title/Abstract] OR "teens"[Title/Abstract] OR "teenage*"[Title/Abstract] OR "juvenile*"[Title/Abstract] OR "minor"[Title/Abstract] OR "minors"[Title/Abstract] OR "underage*"[Title/Abstract] OR "under-age*"[Title/Abstract] OR "school-age*"[Title/Abstract] OR "schoolage*"[Title/Abstract] OR "schoolchild*"[Title/Abstract] OR "schoolgirl*"[Title/Abstract] OR "schoolboy*"[Title/Abstract] OR "young-adult*"[Title/Abstract] OR "young-people*"[Title/Abstract] OR "young-person*"[Title/Abstract] OR "emerging-adult*"[Title/Abstract] OR "emerging-people*"[Title/Abstract] OR "emerging-person*"[Title/Abstract] OR "AYA"[Title/Abstract] OR "AYAs"[Title/Abstract]

13 NOTNLM OR publisher[sb] OR inprocess[sb] OR pubmednotmedline[sb] OR indatareview[sb] OR pubstatusaheadofprint

14 #10 AND #11 AND #12 AND #13

**Embase** <Conception (1974) to 30 August 2023>

1 patient participation/

2 stakeholder engagement/

3 community participation/

4 exp participatory research/

5 (CBPR or participatory-research* or participatory-method* or participatory-approach* or participatory-design or participatory-model*).tw,kf,dq.

6 advisory committee/

7 (advisory-group* or advisory-panel* or advisory-board? or advisory-committee* or focus-group* or expert-group* or ambassador*).tw,kf,dq

8 (co-produc* or coproduc* or co-design* or codesign* or co-research* or coresearch* or co-develop* or codevelop* or co-creation* or cocreation*).tw,kf,dq.

9 (patient* or public or communit* or user* or stakeholder* or consumer* or client* or citizen* or participant*).tw,kf,dq.

10 ((participat* or involv* or consult* or collaborat* or engage* or inform? or informed or informing or contribute or contribution or contributed or contributing or guide? or guiding or guided or guidance* or impact* or input or influenc* or advis* or advocat* or "led" or partner*) adj5 ((research or study or studies or project?) adj3 (design* or model* or program? or programme? or stag* or approach or approaches or process or processes or procedure* or activit* or deliver* or decision? or decision-making or develop* or implement* or execut* or plan? or planning or governance or polic*))).tw,kf,dq.

11 ((participat* or involv* or consult* or collaborat* or engage* or inform? or informed or informing or contribute or contribution or contributed or contributing or guide? or guiding or guided or guidance* or impact* or input or influenc* or advis* or advocat* or "led" or partner*) adj5 ((data or sample? or result? or finding?) adj3 (collect* or analys* or analyz* or disseminat* or "use" or usage or share* or sharing or linkage? or linking or governance or polic*))).tw,kf,dq.

12 1 or 2 or 3 or 4 or 5 or 6 or 7 or 8 or 9

13 10 or 11

14 12 and 13

15 (Patient-and-public-involvement or Patient-and-public-engagement or Public-and-patient-involvement or Public-and-patient-engagement or Patient-public-involvement or Patient-public-engagement or Public-patient-involvement or Public-patient-engagement or Youth-Advisory-Group* or Young-People's-Advisory-Group* or Young-Person's-Advisory-Group* or Young-People's-Action-Group* or Youth-Participatory-Action-Research or Youth-Led-Participatory-Action-Research or YPAG or YPAGs or PPIE or YPAR).tw,kf,dq.

16 14 or 15

17 (health* or biomedical or medic* or clinic*).tw,kf,hw,jw,dq.

18 (newborn* or new-born* or baby or babies or neonat* or neo-nat* or infan* or toddler* or pre-schooler* or preschooler* or kinder or kinders or kindergarten* or kinder-aged or boy or boys or girl or girls or child or children or childhood or youngster* or kid or kids or preadolescen* or pre-puberty or prepuberty or pre-pubescen* or prepubescen* or adolescen* or pediatric* or paediatric* or youth or youths or teen or teens or teenage* or juvenile* or minor or minors or underage* or under-age* or school-age* or schoolage* or schoolchild* or schoolgirl* or schoolboy* or young-adult* or young-people* or young-person* or emerging-adult* or emerging-people* or emerging-person* or AYA or AYAs).tw,kf,hw,dq.

19 (systematic-review or rapid-review or meta-analysis or meta-synthesis or meta-review or literature-review or scoping-review or umbrella-review or narrative-review).tw,kf,hw,dq. or "review"/ or "systematic review"/

20 16 and 17 and 18 and 19
