# Supplementary material for: Involvement of children and young people in the conduct of health research: A rapid umbrella review
Source: Health Expect. 2024 Jun 6;27(3):e14081. doi: 10.1111/hex.14081 (PMC11156690; doi:10.1111/hex.14081)
Supplement: Supplementary file 2 — Supporting information. [file HEX-27-e14081-s003.docx]

**Appendix 2:** Methods of Involvement by Age Group (n, % of studies with available data).

| Method of Involvement | All (n=600 studies) CYP (and/or their representatives) | CYP alone (n=397 studies) ≤24 years of age; no representatives | Children alone (n=49 studies) ≤12 years of age; no representatives |
| --- | --- | --- | --- |
| Verbal Methods | 488 (81%) | 300 (76%) | 39 (80%) |
| Interviews | 211 (35%) | 118 (30%) | 13 (27%) |
| Focus Groups | 282 (47%) | 162 (41%) | 20 (41%) |
| Advisory Groups / Co-researchers | 142 (24%) | 103 (27%) | 12 (24%) |
| Storytelling | 1 (0.2%) | 1 (0.3%) | 0 (0%) |
| Written Methods | 90 (15%) | 61 (15%) | 5 (10%) |
| Questionnaires | 87 (15%) | 59 (15%) | 5 (10%) |
| Diary writing | 3 (1%) | 2 (1%) | 0 (0%) |
| Story-writing | 0 (0%) | 0 (0%) | 0 (0%) |
| Visual Methods | 163 (27%) | 136 (34%) | 16 (33%) |
| Drawing | 37 (6%) | 25 (6%) | 9 (18%) |
| Video/film making | 9 (2%) | 6 (2%) | 1 (2%) |
| Photography | 29 (5%) | 19 (5%) | 4 (8%) |
| Photovoice | 87 (15%) | 80 (20%) | 3 (6%) |
| Mapping activities | 14 (2%) | 11 (3%) | 0 (0%) |
| Active Methods | 18 (3%) | 16 (4%) | 6 (12%) |
| Play (e.g. Puppetry) | 7 (1%) | 7 (2%) | 4 (8%) |
| Drama or Role-play | 10 (2%) | 8 (2%) | 2 (4%) |
| Music or Dance | 2 (0.3%) | 2 (1%) | 0 (0%) |
| Other | 24 (4%) | 24 (6%) | 0 (0%) |
| Combination Methods | 170 (28%) | 128 (32%) | 17 (35%) |
| Mosaic Approach | 2 (0%) | 0 (0%) | 0 (0%) |
| Photo/Image Elicitation | 4 (1%) | 1 (0.3%) | 0 (0%) |
| Draw-and-write | 2 (0.3%) | 2 (1%) | 2 (4%) |

*CYP = Children and Young People.*
