# Supplementary material for: Involvement of children and young people in the conduct of health research: A rapid umbrella review
Source: Health Expect. 2024 Jun 6;27(3):e14081. doi: 10.1111/hex.14081 (PMC11156690; doi:10.1111/hex.14081)

**Appendix 3.** Risk of Bias of Systematic Reviews (ROBIS) Assessment

| **Review** | **Phase 2 (Domains 1-4)** | | | | **Phase 3** |
| --- | --- | --- | --- | --- | --- |
|  | 1. Study eligibility criteria | 2. Identification and selection of studies | 3. Data collection and study appraisal | 4.Synthesis and findings | Risk of Bias in the review |
| Bailey, 2015 | 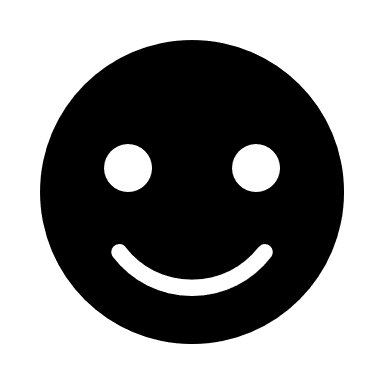 | 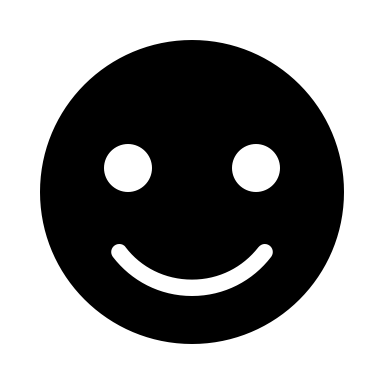 | 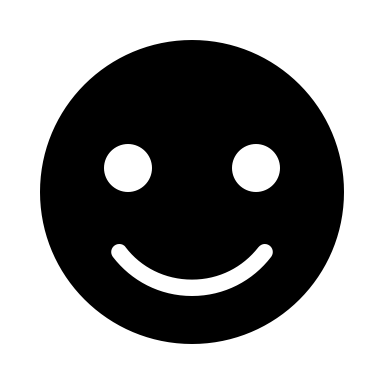 | 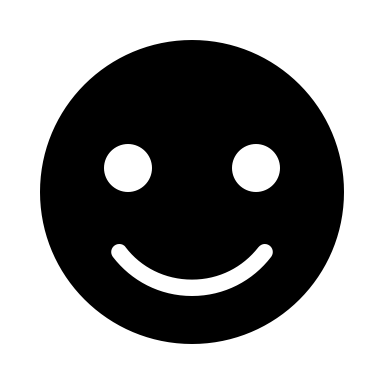 | 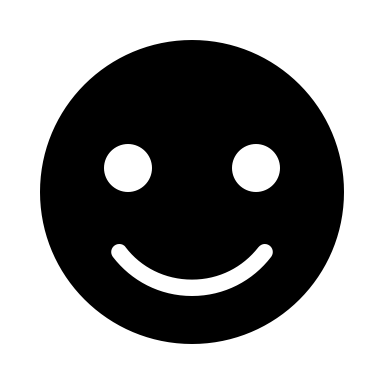 |
| Bakhtiar, 2023 | 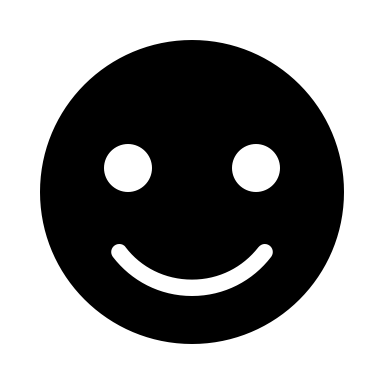 | 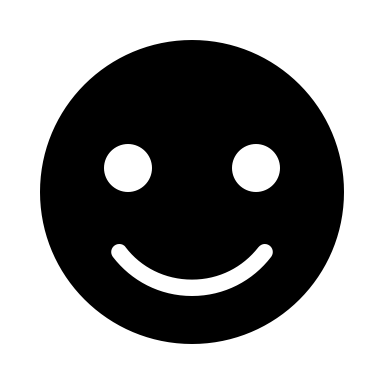 | 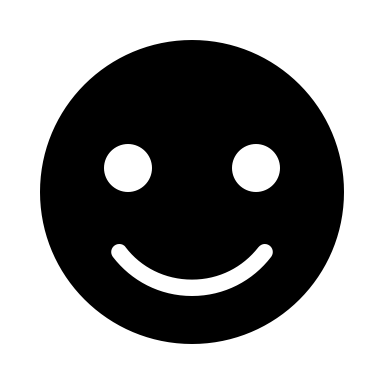 | 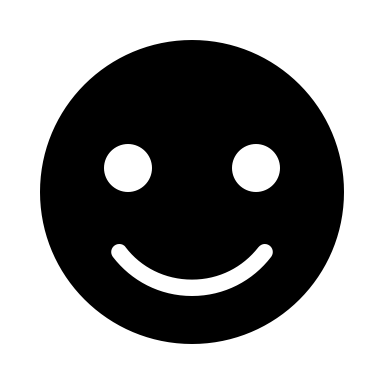 | 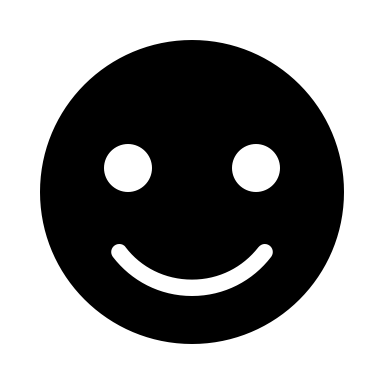 |
| Branquinho, 2020 | 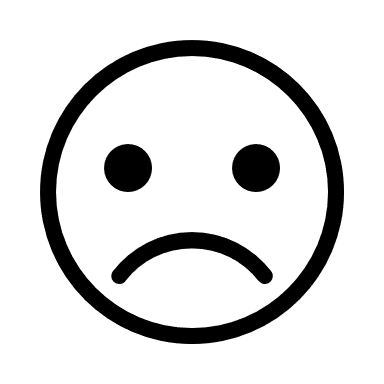 | 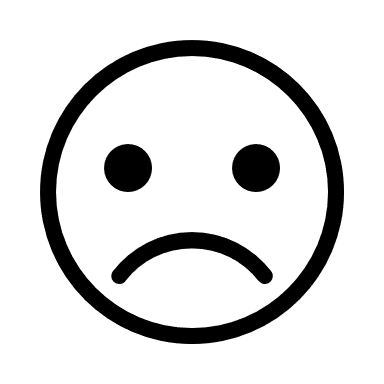 | 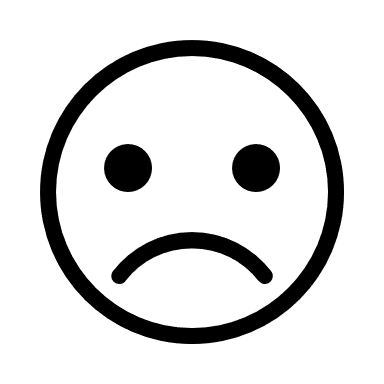 | 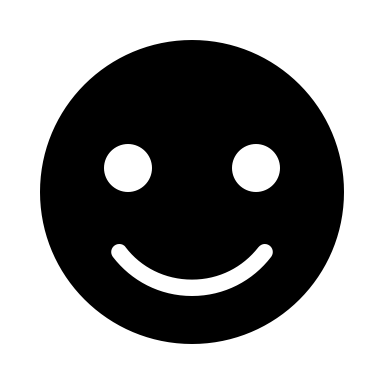 | 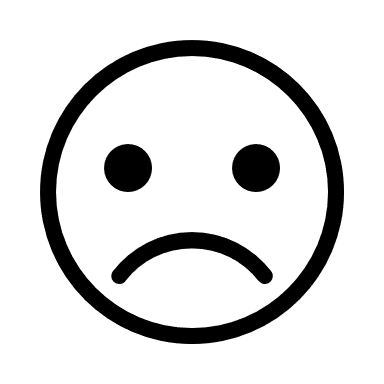 |
| Fountain, 2021 | 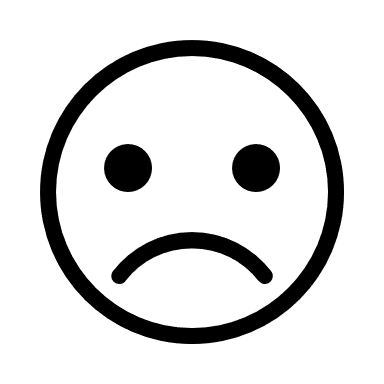 | 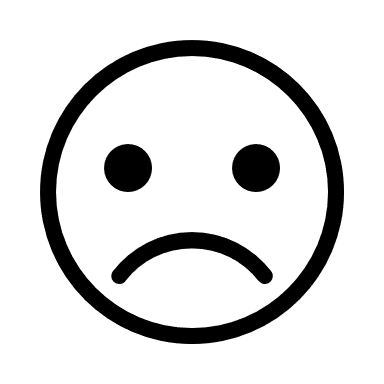 | 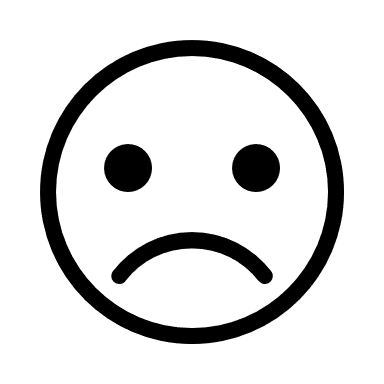 | 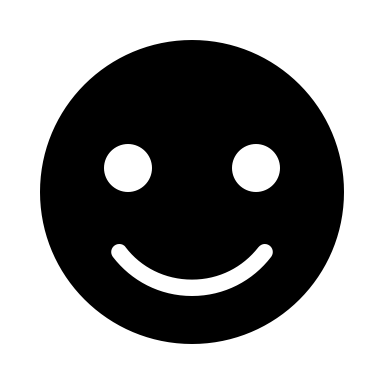 | 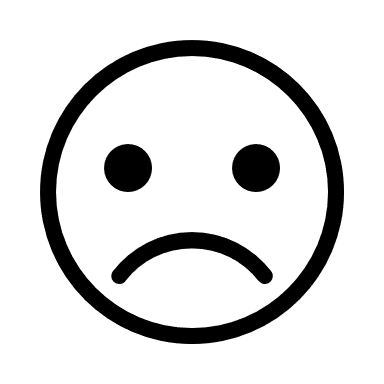 |
| Freire, 2022 | 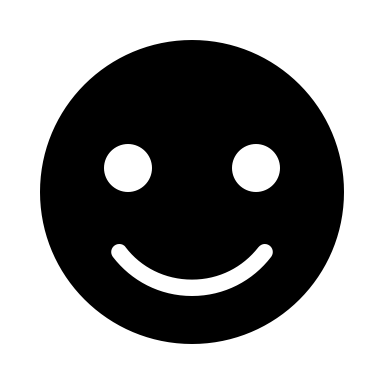 | 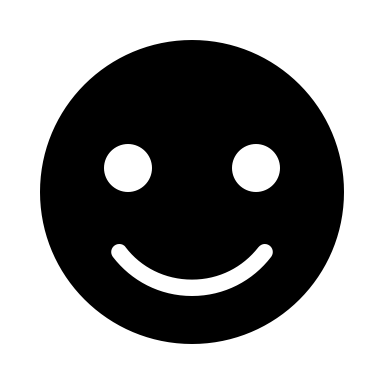 | 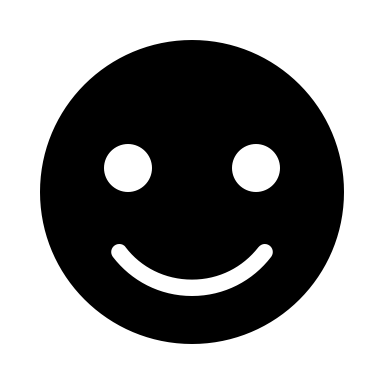 | 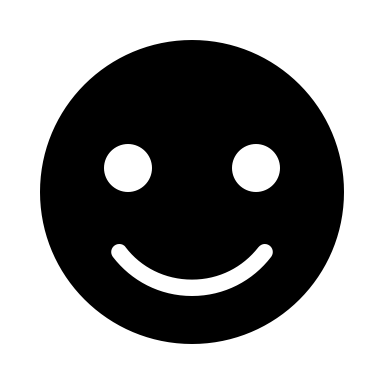 | 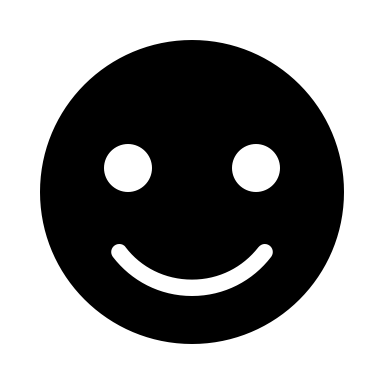 |
| Gibbs, 2020 | 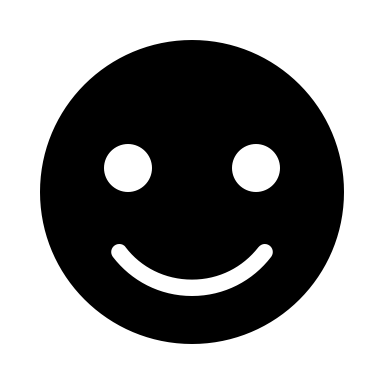 | 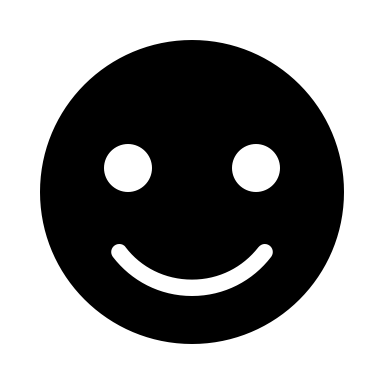 | 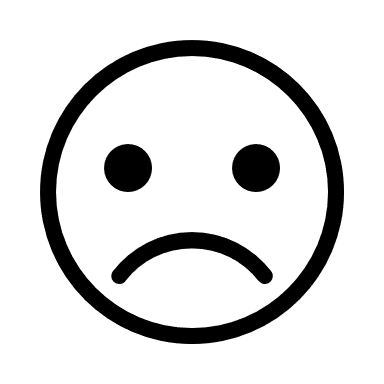 | 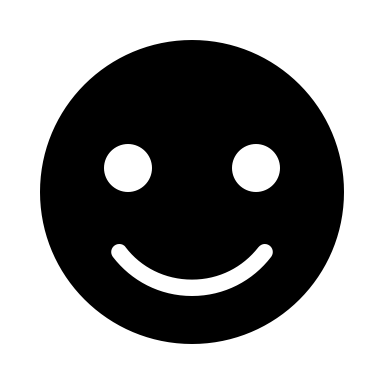 | 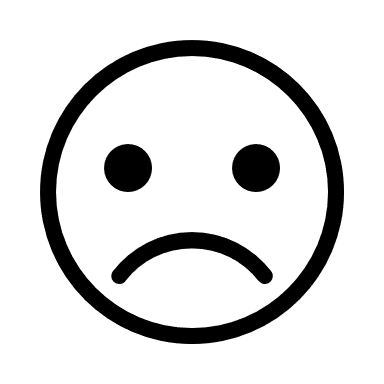 |
| Haijes, 2016 | 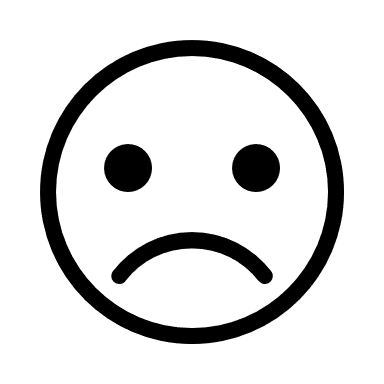 | 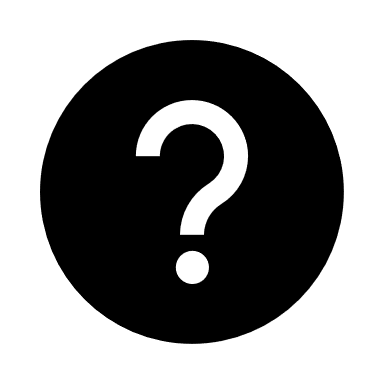 | 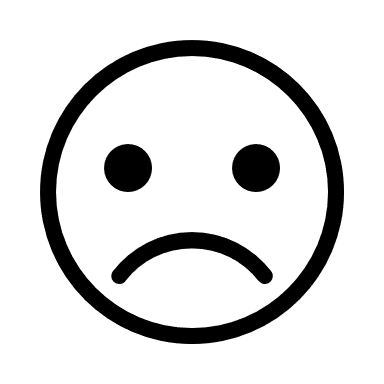 | 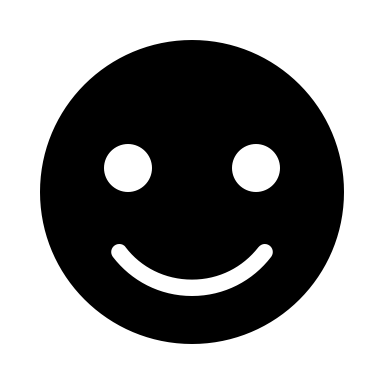 | 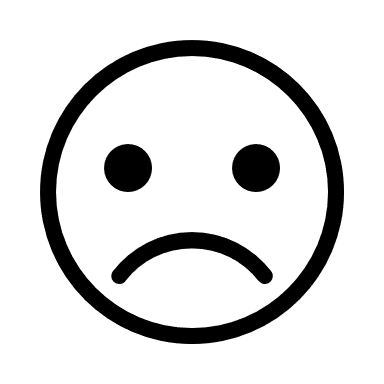 |
| McCabe, 2023 | 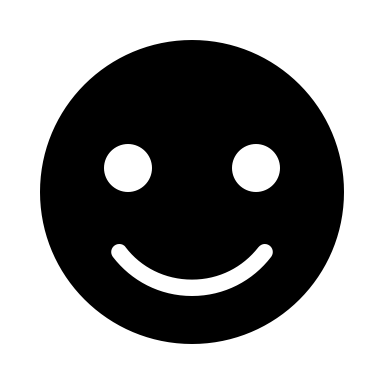 | 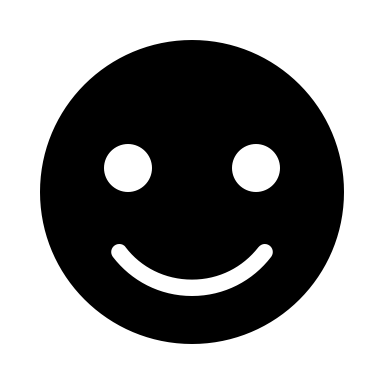 | 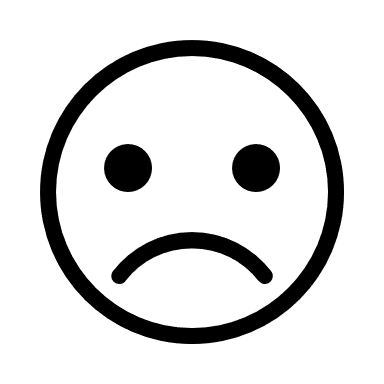 | 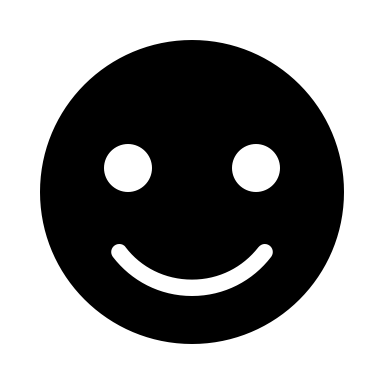 | 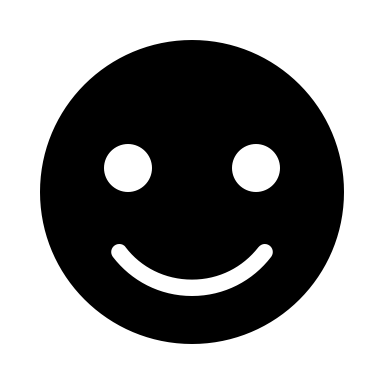 |
| McNeill, 2021 | 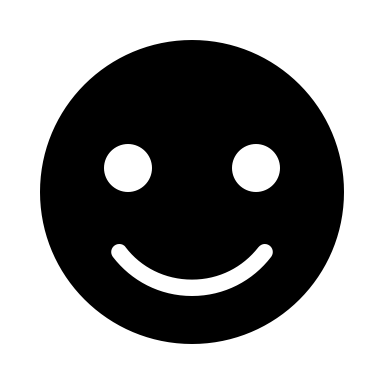 | 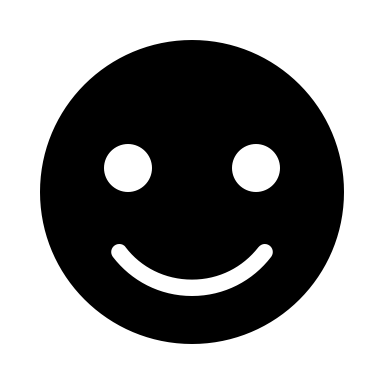 | 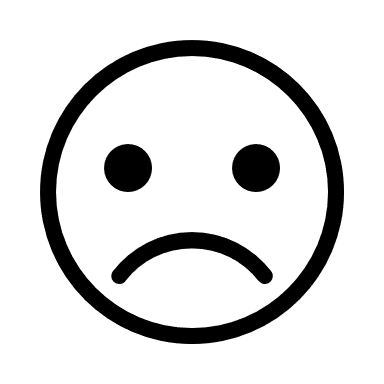 | 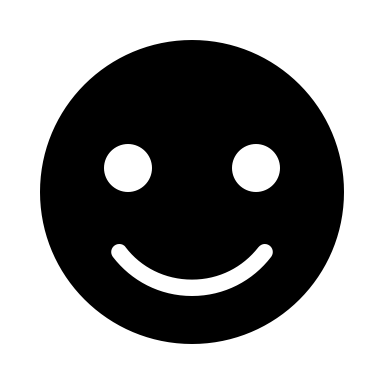 | 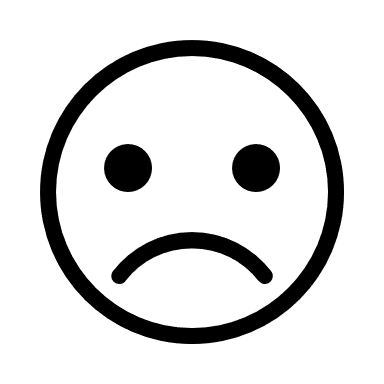 |
| Nathan, 2023 | 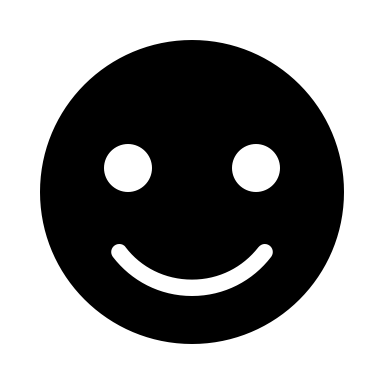 | 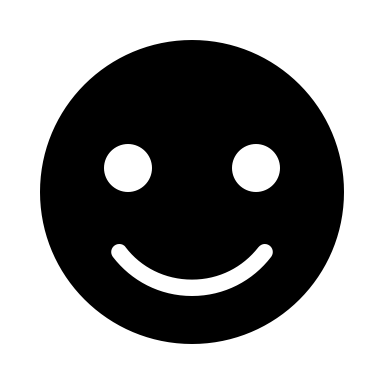 | 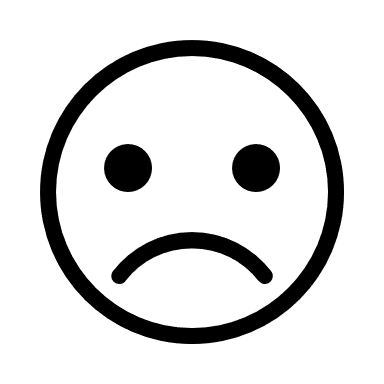 | 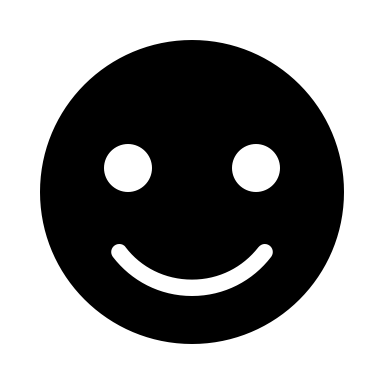 | 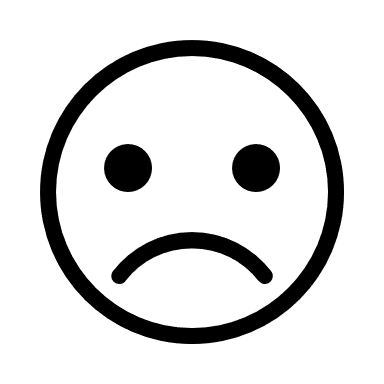 |
| Nortvedt, 2022 | 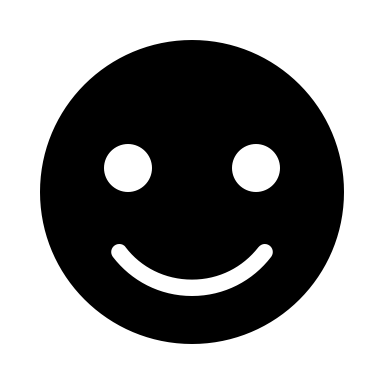 | 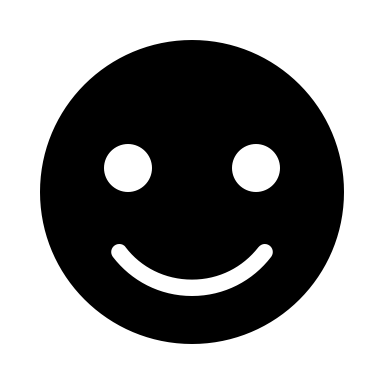 | 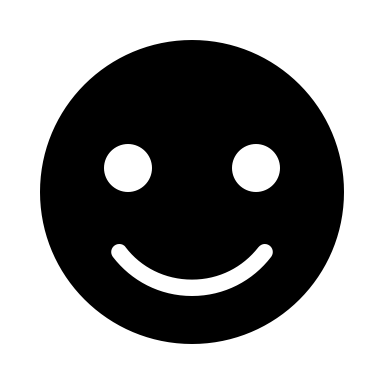 | 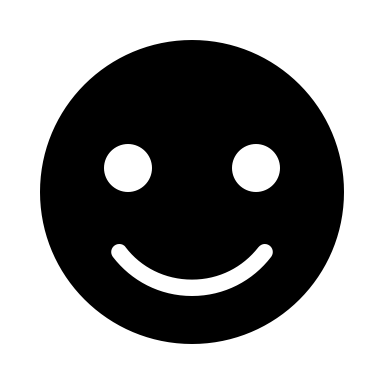 | 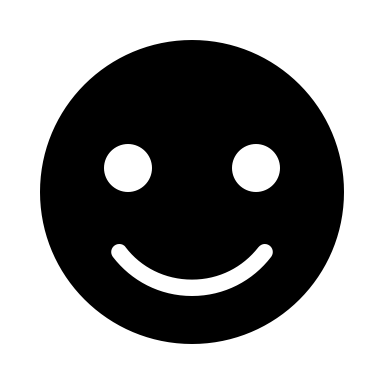 |
| Valdez, 2020 | 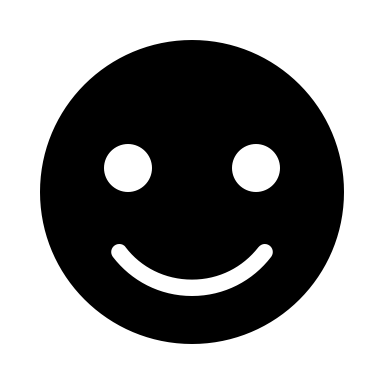 | 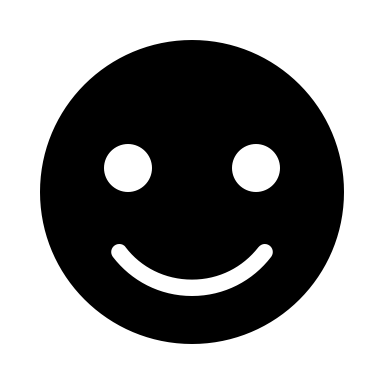 | 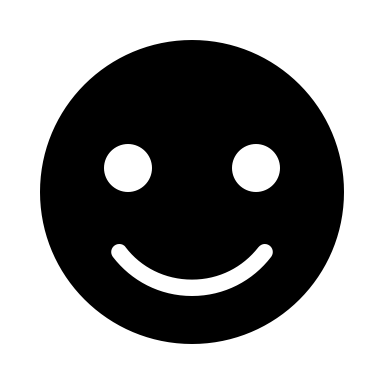 | 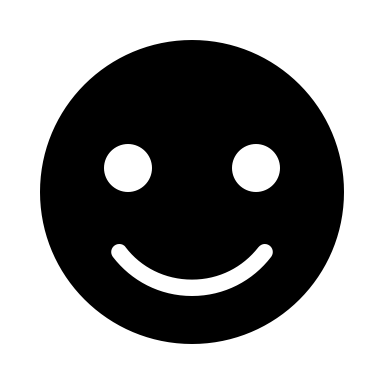 | 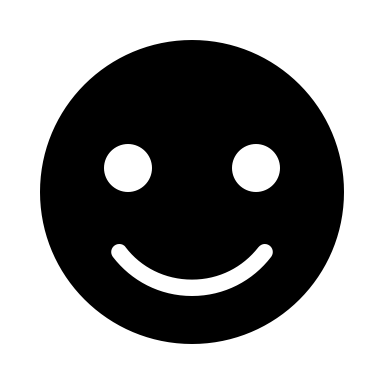 |

= Low risk
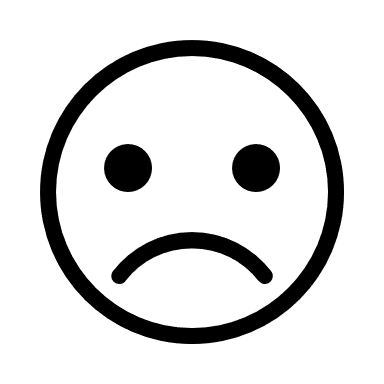
=High risk;
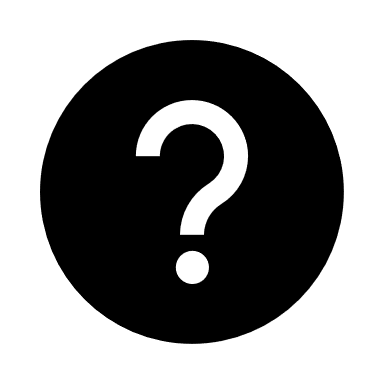
= Unclear risk


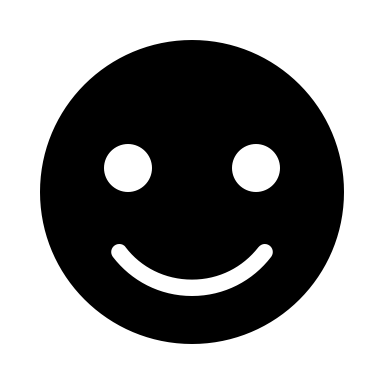

Supplement: Supplementary file 3 — Supporting information. [file HEX-27-e14081-s002.docx]
